# Association of radiation-induced normal tissue toxicity with a high genetic risk for rheumatoid arthritis

**DOI:** 10.1093/jnci/djae349

**Published:** 2025-01-06

**Authors:** Alan McWilliam, Deborah Marshall, Sarah L Kerns, Gillian C Barnett, Ana Vega, Thodori Kapouranis, Miguel E Aguado Barrera, Barbara Avuzzi, David Azria, Jenny Chang-Claude, Ananya Choudhury, Carla Coedo Costa, Alison Dunning, Marie-Pierre Farcy-Jacquet, Corinne Faivre-Finn, Sara Gutiérrez-Enríquez, Olivia Fuentes-Ríos, Antonio Gómez Caamaño, Maarten Lambrecht, Carlos López Pleguezuelos, Tiziana Rancati, Tim Rattay, Dirk de Ruysscher, Petra Seibold, Elena Sperk, Christopher Talbot, Adam Webb, Liv Veldeman, Barry S Rosenstein, Catharine M L West, REQUITE Consortium, P Stegmaier, J Claßen, T Schnabel, Anusha Müller, Irmgard Helmbold, Rebecca Elliott

**Affiliations:** Division of Cancer Sciences, University of Manchester, Manchester, United Kingdom; The Christie NHS Foundation Trust, Manchester, United Kingdom; Department of Radiation Oncology, Icahn School of Medicine at Mount Sinai, New York, NY, United States; Department of Population Health Science and Policy, Icahn School of Medicine at Mount Sinai, New York, NY, United States; Medical College of Wisconsin, Milwaukee, WI, United States; Oncology Centre, Cambridge University Hospitals NHS Foundation Trust, Cambridge, United Kingdom; Instituto de Investigación Sanitaria de Santiago de Compostela (IDIS), Santiago de Compostela, Spain; Fundación Pública Galega de Medicina Xenómica (FPGMX), Santiago de Compostela, Spain; Biomedical Network on Rare Diseases (CIBERER), Spain; Department of Population Health Science and Policy, Icahn School of Medicine at Mount Sinai, New York, NY, United States; Instituto de Investigación Sanitaria de Santiago de Compostela (IDIS), Santiago de Compostela, Spain; Fundación Pública Galega de Medicina Xenómica (FPGMX), Santiago de Compostela, Spain; Department of Radiation Oncology, Fondazione Istituto di ricovero e cura a carattere scientifico (IRCCS) Istituto Nazionale dei Tumori, Milan, Italy; Federation Universitaire d’Oncologie Radiothérapie d’Occitanie Méditerranée, Univ Montpellier, INSERM U1194 IRCM, Institut du Cancer Montpellier (ICM), Montpellier, France; German Cancer Research Center (DKFZ), Division of Cancer Epidemiology, Heidelberg, Germany; University Cancer Center Hamburg, University Medical Center Hamburg-Eppendorf, Hamburg, Germany; Division of Cancer Sciences, University of Manchester, Manchester, United Kingdom; The Christie NHS Foundation Trust, Manchester, United Kingdom; Instituto de Investigación Sanitaria de Santiago de Compostela (IDIS), Santiago de Compostela, Spain; Fundación Pública Galega de Medicina Xenómica (FPGMX), Santiago de Compostela, Spain; Escola de Doutoramento Interacional, Universidade de Santiago de Compostela, Santiago de Compostela, Spain; Centre for Cancer Genetic Epidemiology, Strangeways Research Laboratory, University of Cambridge, Cambridge, United Kingdom; Federation Universitaire d’Oncologie Radiothérapie d’Occitanie Méditerranée, Institut du Cancer Du Gard (ICG), CHU Carémeau, Nîmes, France; Division of Cancer Sciences, University of Manchester, Manchester, United Kingdom; The Christie NHS Foundation Trust, Manchester, United Kingdom; Hereditary Cancer Genetics Group, Vall d’Hebron Institute of Oncology (VHIO), Vall d’Hebron Barcelona Hospital Campus, Barcelona, Spain; Instituto de Investigación Sanitaria de Santiago de Compostela (IDIS), Santiago de Compostela, Spain; Fundación Pública Galega de Medicina Xenómica (FPGMX), Santiago de Compostela, Spain; Instituto de Investigación Sanitaria de Santiago de Compostela (IDIS), Santiago de Compostela, Spain; Department of Radiation Oncology, Complexo Hospitalario Universitario de Santiago, Servizo Galego de Saúde (SERGAS), Santiago de Compostela, Spain; KU Leuven, Leuven, Belgium; Instituto de Investigación Sanitaria de Santiago de Compostela (IDIS), Santiago de Compostela, Spain; Fundación Pública Galega de Medicina Xenómica (FPGMX), Santiago de Compostela, Spain; Escola de Doutoramento Interacional, Universidade de Santiago de Compostela, Santiago de Compostela, Spain; Data Science Unit, Fondazione IRCCS Istituto Nazionale dei Tumori, Milan, Italy; Leicester Cancer Research Centre, University of Leicester, Leicester, United Kingdom; Maastro Clinic, Maastricht, The Netherlands; German Cancer Research Center (DKFZ), Division of Cancer Epidemiology, Heidelberg, Germany; Department of Radiation Oncology, Universitätsmedizin Mannheim, Medical Faculty Mannheim, University of Heidelberg, Mannheim, Germany; Department of Genetics & Cancer Sciences, University of Leicester, Maastrcht, United Kingdom; Department of Genetics & Cancer Sciences, University of Leicester, Maastrcht, United Kingdom; Department of Radiation Oncology, Ghent University Hospital, Ghent, Belgium; Department of Human Structure and Repair, Ghent University, Ghent, Belgium; Department of Radiation Oncology, Icahn School of Medicine at Mount Sinai, New York, NY, United States; Department of Genetics and Genomic Sciences, Icahn School of Medicine at Mount Sinai, New York, NY, United States; Division of Cancer Sciences, University of Manchester, Manchester, United Kingdom; The Christie NHS Foundation Trust, Manchester, United Kingdom; Translational Radiobiology Group, Manchester, United Kingdom

## Abstract

**Background:**

Overlapping genes are involved with rheumatoid arthritis (RA) and DNA repair pathways. Therefore, we hypothesized that patients with a high polygenic risk score for RA will have an increased risk of radiotherapy toxicity given the involvement of DNA repair.

**Methods:**

Primary analysis was performed on 1494 prostate cancer, 483 lung cancer, and 1820 breast cancer patients assessed for development of radiotherapy toxicity in the REQUITE (validating pREdictive models and biomarkers of radiotherapy toxicity to reduce side effects and improve QUalITy of lifE in cancer survivors) study. Validation cohorts were available from the Radiogenomics Consortium. All patients had undergone curative-intent radiotherapy and were assessed prospectively for toxicity. Germline genomic data was available for all patients, allowing a polygenic risk score to be calculated using 101 RA risk variants. Polygenic risk score was analyzed as a continuous variable and with a more than 90th percentile cutoff. Associations with acute and late standardized total average toxicity (STAT) scores and individual toxicity endpoints were analyzed in multivariable models with preselected adjustment variables.

**Results:**

Increasing polygenic risk score for RA did not increase the risk of STAT-acute or STAT-late in any cohort. There was an increased risk of late esophagitis in the lung cancer cohort (coefficient = 0.018, *P* = .01), however this was not validated (*P* = .79). No individual acute or late toxicity endpoints were statistically significantly associated with polygenic risk score for the prostate or breast cohorts. No statistically significant results were found in the validation cohorts in multivariable models.

**Conclusions:**

Patients with a high genetic risk for RA do not show increased levels of toxicity after radiotherapy suggesting treatment planning does not need to be modified for such patients.

## Introduction

Rheumatoid arthritis (RA) is a systemic autoimmune disease involving persistent joint inflammation, which results in cartilage and bone damage. There is conflicting evidence in the literature regarding the potential connection between RA and increased risk and severity of radiation-induced toxicities.^1^ RA may also be associated with increased radiosensitivity. RA is a complex disease, and genome-wide association studies (GWAS) have identified multiple predisposing risk loci.^2-4^ This polygenicity of RA results in complex and unique interactions between genes for each individual, leading to variable disease characteristics.

One potential mechanism is the multiple potential interactions between the genes involved in RA and individual radiosensitivity, which may result in an increased risk and severity of toxicity after radiotherapy. A full description of these overlapping genes is available in the systematic review by Liebenberg et al.^1^ Briefly, there are overlapping genes involved in RA and DNA damage responses; particularly, the *ATM* and *RAD51B* genes are identified in RA GWAS and are important in DNA damage response pathways.^2-6^*ATM* is active in DNA double-strand break repair via nonhomologous end joining and in V(D)J recombination.^7^^,^^8^ More generally, there are potential interactions between the risk loci for immunodeficiencies and genes involved in DNA repair.^6^^,^^9^^,^^10^ Additionally, RA involves dysregulation of the immune system and inflammatory processes,^9^^,^^11^ which have potential extra-articular effects (ie, inflammation outside the joint tissues).^11^ Inflammation and the immune response are also involved in radiation-induced normal tissue toxicity.^12^^,^^13^ This evidence strongly supports the hypothesis that individuals with RA will have an increased risk of normal tissue complications postradiotherapy, prompting concern over treating such patients with a standard radiotherapy plan and normal tissue dose constraints. However, there are conflicting reports and limited evidence in the literature to confirm or refute the hypothesis.^1^^,^^14-19^ Further complicating the clinical scenario, as other polygenic diseases, some people will carry risk variants for RA but will not manifest disease or receive a diagnosis. For these patients, their genetic predisposition to RA and possible increased risk of radiotherapy toxicity would not be known to the treating radiation oncologist. To this end, we investigate the relationship between individuals’ underlying polygenic risk score for RA and their risk of radiotherapy-related toxicity. This approach will elucidate if it is an individual’s genetic predisputation to RA, which drives these observed outcomes.

In this study, we focus on radiotherapy-related toxicity and hypothesize that individuals with an increased genetic predisposition to RA will have an increased risk of normal tissue toxicity. To test the hypothesis, we generated polygenetic risk scores for RA for individual patients treated with radiotherapy for prostate, breast, or lung cancer. Our aim was to test whether patients at high genetic risk of RA have an increased risk of normal tissue toxicity after receiving curative-intent radiotherapy.

## Methods

The Strengthening the Reporting of Genetic Association Studies in Radiogenomics recommendations were followed during the conceptualization, analysis, and reporting of this study.^20^

### Patient cohorts

The primary discovery cohort in this analysis was from REQUITE (validating pREdictive models and biomarkers of radiotherapy toxicity to reduce side effects and improve QUalITy of lifE in cancer survivors), a multicenter, international prospective study with standardized longitudinal data and blood sample collection.^21^^,^^22^ Data were available from 1760 prostate, 2059 breast, and 530 lung cancer patients treated with radiotherapy alone or as part of a curative-intent treatment. Normal tissue toxicity data were collected prospectively for all patients up to 2 years postradiotherapy, scored according to the Common Terminology Criteria for Adverse Effects scale, v4.0, for health-care professional and patient-reported toxicities. Germline DNA was genotyped using Illumina OncoArray-500K BeadChips and imputed using the 1000 Genomes Project Phase 3.

Independent validation datasets were available from each cohort and fully described in the Supplementary Material S1. Briefly, prostate validation was available from RT01^23^ (ISRCTN47772397) and CHHiP^24^ (ISRCTN97182923) trials and was approved by the Cambridge South research ethics committee (05/Q0108/365). Other prostate cohorts were available through data sharing agreements through the international Radiogenomics Consortium.^25^ The breast validation was available from recruited participants enrolled in the Cambridge Intensity Modulated Radiation Therapy (IMRT) Trial (ISRCTN21474421).^26^ The lung validation cohort was available from RADIOGEN-recruited participants treated at the Clinical University Hospital of Santiago de Compostela, Spain, and was approved by the Galician Ethical Committee.^27^^,^^28^ Toxicity definitions for all cohorts are included in Table S1. All participants had given their written consent for their data to be used for research (Methods S1).

### Toxicity

Acute toxicities were defined as the maximum reported toxicity within 3 months of radiotherapy and late toxicities as the maximum reported after 3 months up to 2 years postradiotherapy (the primary endpoint in REQUITE). Where baseline measures (start of radiotherapy) were available, they were accounted for by calculating a delta measure, defining the increase in reported toxicity due to radiotherapy.

The following acute toxicities were selected in each cohort: prostate (urinary frequency, nocturia, urinary urgency, hematuria, urinary incontinence, decreased stream, dysuria, gastrointestinal incontinence, diarrhea, gastrointestinal urgency, and tenesmus); breast (erythema and ulceration); and lung (cough, dyspnea, pneumonitis, dysphagia, and esophagitis). The following late toxicities were selected in each cohort: prostate (proctitis, rectal bleeding, hematuria, urinary frequency, and urinary retention); breast (telangiectasia, edema, induration, pigmentation, and atrophy); and lung (cough, dyspnea, pneumonitis, dysphagia, and esophagitis).

Standardized total average toxicity (STAT) scores were calculated following the methodology defined by Barnett et al.^29^ STAT pools data from several toxicity endpoints into a measure of overall toxicity experienced by each patient. The acute and late toxicities defined above for each cohort were used to calculate STAT-acute and STAT-late for each patient within a cohort.

### Calculation of polygenic risk score

The risk alleles for RA were defined from the GWAS study by Okada et al.;^2^ 101 risk alleles were defined. The polygenic risk score was calculated by summing the dosage of each risk allele. A weighted polygenic risk score was calculated by weighting the dosage by the odds ratio presented in the GWAS. Distributions of polygenic risk score and weighted polygenic risk score were plotted and tested for normality. Differences in polygenic risk score and weighted polygenic risk score for patients with and without a documented diagnosis of RA in the datasets were tested using a Mann–Whitney *U* test.

### Statistical analysis

Preselected clinical variables were defined for inclusion in multivariable models. These were prostate (age, diabetes, prior surgery, hormone therapy, prescription dose [converted to a biologically effective dose using an alpha-beta ratio = 10 Gy]); lung (sex, age, smoking status, radiotherapy technique, fev1, v20 lungs, v35 esophagus, prescription dose [biologically effective dose, using an alpha/beta = 10 Gy]), chronic obstructive pulmonary disease; and breast (age, smoker status, cardiovasculature disease, body mass index, breast volume, diabetes, postoperative breast infection, breast boost).

Associations of the polygenic risk score and weighted polygenic risk score with STAT-acute and STAT-late were tested with univariable and multivariable linear regression. Polygenic risk score and weighted polygenic risk score were dichotomized at the 90th percentile to investigate associations for those individuals at highest genetic risk. Associations were considered statistically significant if *P* value was less than .05. Individual acute and late toxicity endpoints were analyzed with multivariable linear regression, adjusting for the variables described. Multiplicity was accounted for with a Bonferroni correction for the number of toxicity endpoints included within each treatment site. All statistical analyses were performed with R version 4.1.1. Validation cohorts were analyzed independently with matched clinical variables and analysis procedure with Bonferroni corrections applied for testing statistical significance . In addition, multivariable regressions by RA diagnosis phenotype were analyzed independently.

## Results

A total of 1494 prostate, 1820 breast, and 483 lung cancer patients were selected from the REQUITE cohort for analysis. These patients were of European ancestry and with sufficient genetic information available to calculate the RA polygenic risk score and with toxicity information available to calculate STAT-acute and STAT-late. Patient characteristics for all patient groups are included in Table S2. Table S3 details the incidence of each acute and late toxicity for each cohort. Histograms showing the distribution of STAT-acute and STAT-late are included in Figure S1 for the REQUITE cohort. Histograms show a right-sided skew as expected because of a small number of patients experiencing more severe toxicity.

**Figure 1.**
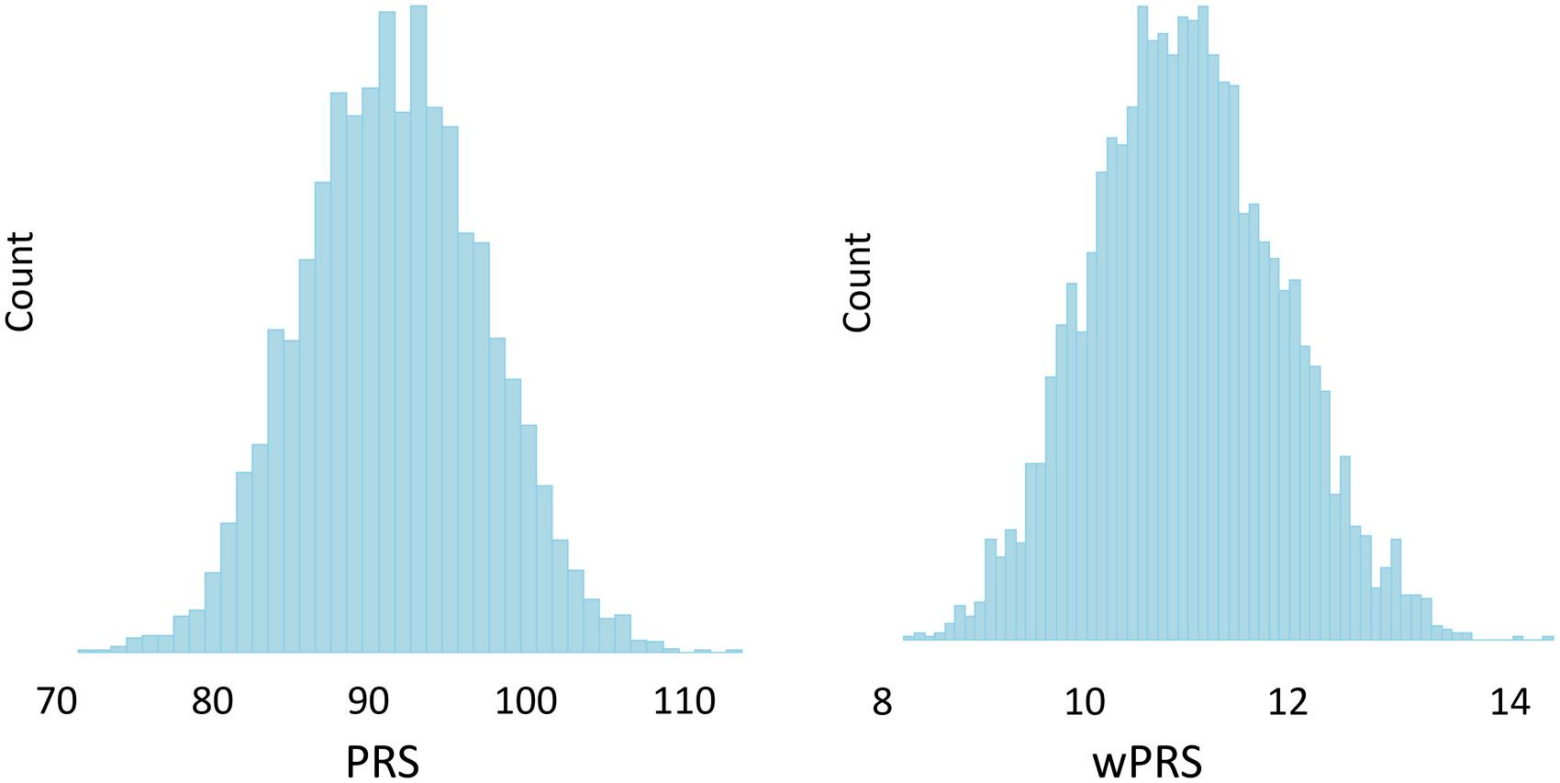
The distributions of polygenic risk score and the weighted polygenic risk score for the REQUITE cohort, calculated using the 101 risk loci identified by Okadaet al.^2^ PRS = polygenic risk score; wPRS = weighted polygenic risk score.

**Figure 2.**
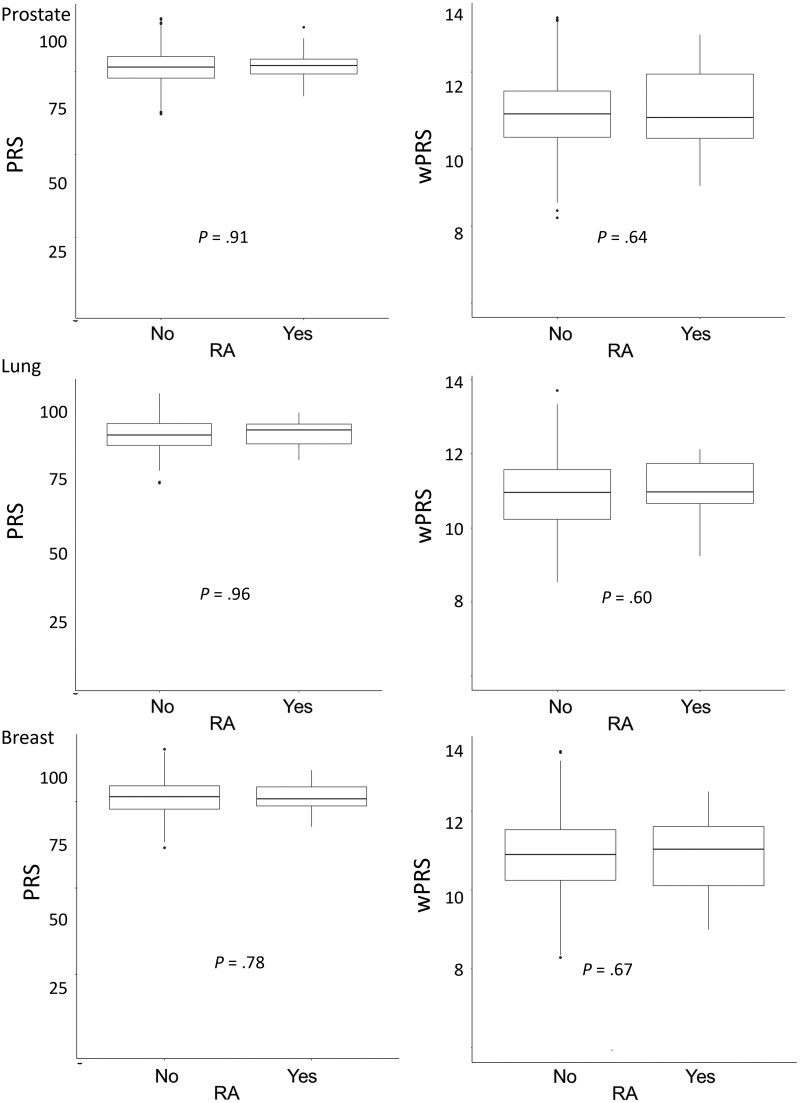
Box and whisker plots showing no differences in the calculated polygenic risk score and weighted polygenic risk score between individuals who have a documented diagnosis of rheumatoid arthritis in the REQUITE cohort. Diagnosis of rheumatoid arthritis in the respective cohorts is prostate 29 of 1494; lung 16 of 483; and breast 55 of 1820. PRS = polygenic risk score; wPRS = weighted polygenic risk score; RA = rheumatoid arthritis.

Polygenic risk score and weighted polygenic risk score were normally distributed across the whole REQUITE cohort (Figure 1) and for the individual cohorts for the 3 treatment sites. Figure 2 shows that there were no statistically significant differences in polygenic risk score or weighted polygenic risk score for patients with or without a documented diagnosis of RA in each treatment group. Table 1 shows the results for STAT-acute and STAT-late, no statistically significant associations were found with the polygenic risk score used as a continuous variable or dichotomized at the 90th percentile, either in univariable or multivariable analysis. Univariable and multivariable results for weighted polygenic risk score are included in Table S4; these models showed no significant association of STAT-acute or STAT-late with weighted polygenic risk score.

**Table 1.** Univariable and multivariable analysis for acute standardized total average toxicity and late standardized total average toxicity with polygenic risk score as a continuous variable and dichotomized at the 90th percentile in the REQUITE cohort

	Acute standardized total average toxicity (polygenic risk score continuous)				Late standardized total average toxicity (polygenic risk score 90th percentile)			
	Univariable		Multivariable^a^		Univariable		Multivariable^a^	
	Beta (95% CI)	*P*	Beta (95% CI)	*P*	Beta (95% CI)	*P*	Beta (95% CI)	*P*
Prostate	−0.003 (−0.007 to 0.002)	.29	−0.002 (−0.007 to 0.002)	.29	−0.064 (−0.015 to 0.025)	.16	−0.061 (−0.150 to 0.026)	.17
Lung	−0.006 (−0.016 to 0.005)	.30	−0.005 (−0.015 to 0.005)	.36	−0.045 (−0.242 to 0.151)	.65	−0.010 (−0.020 to 0.180)	.92
Breast	0.002 (−0.010 to 0.007)	.72	−0.004 (−0.011 to 0.004)	.33	0.003 (−0.151 to 0.156)	.97	−0.017 (−0.170 to 0.133)	.82

	Late standardized total average toxicity (polygenic risk score continuous)				Late standardized total average toxicity (polygenic risk score 90th percentile)			
	Univariable		Multivariable^a^		Univariable		Multivariable^a^	
	Beta (95% CI)	*P*	Beta (95% CI)	*P*	Beta (95% CI)	*P*	Beta (95% CI)	*P*
Prostate	0.003 (−0.003 to 0.008)	.35	0.003 (−0.004 to 0.009)	.36	0.064 (−0.095 to 0.108)	.20	0.006 (−0.096 to 0.109)	.91
Lung	0.006 (−0.004 to 0.018)	.26	0.006 (−0.004 to 0.017)	.26	0.167 (−0.034 to 0.368)	.10	0.145 (−0.052 to 0.348)	.14
Breast	0.001 (−0.004 to 0.004)	.88	0.001 (−0.003 to 0.004)	.82	−0.011 (−0.095 to 0.072)	.79	0.006 (−0.076 to 0.087)	.89

Abbreviations: CI = confidence interval; IMRT = Intensity Modulated Radiation Therapy.

a For the multivariable analysis, the following adjustment variables were included: prostate (age at radiotherapy, diabetes, prior surgery, hormone therapy, prescription dose [converted to biologically effective dose]); lung (sex, age at radiotherapy, prior or current smoker, radiotherapy technique [3-dimensional conformal, arc, IMRT, tomotherapy, stereotactic radiotherapy], fev1, v20 lungs, v35 esophagus, prescription dose [converted to biologically effective dose], diagnosis of chronic obstructive pulmonary disease; and breast (age at radiotherapy, prior or current smoker, history of cardiovasculature disease, body mass index, breast volume, diagnosis of diabetes, postoperative breast infection, received breast boost).

Multivariable results for individual acute toxicity endpoints are included in Table 2. For patients with prostate cancer, rectal bleeding showed a statistically significant association for patients in the top 10th percentile of weighted polygenic risk score after Bonferroni correction (coefficient = −0.30; 95% confidence interval [CI] = −0.48 to −0.11; *P* = .002; Bonferroni correction for 14 acute endpoints, significance level *P* = .004). This result suggests a high genetic risk for RA is protective for risk of rectal bleeding. No acute toxicity endpoints were found significant for patients with breast or lung cancer after Bonferroni correction.

**Table 2.** Multivariable analysis for individual acute toxicity endpoints with polygenic risk score and weighted polygenic risk score as continuous variables and dichotomized at the 90th percentile in the REQUITE cohort^a^

Toxicity		Continuous variables		90th percentile cut	
		Beta (95% CI)	*P*	Beta (95% CI)	*P*
Prostate	Urinary frequency	PRS: −0.001 (−0.010 to 0.008) wPRS: −0.038 (−0.096 to 0.020)	.85 .20	PRS: −0.038 (−0.096 to 0.020) wPRS: −0.089 (−0.264 to 0.086)	.20 .32
	Nocturia	PRS: −0.003 (−0.012 to 0.006) wPRS: −0.034 (−0.089 to 0.023)	.47 .25	PRS: −0.033 (−0.089 to 0.023) wPRS: −0.007 (−0.176 to 0.162)	.25 .93
	Urinary urgency	PRS: −0.007 (−0.020 to 0.006) wPRS: −0.059 (−0.144 to 0.026)	.31 .18	PRS: −0.059 (−0.144 to 0.026) wPRS: −0.046 (−0.300 to 0.208)	.17 .72
	Hematuria	PRS: −0.001 (−0.002 to 0.001) wPRS: −0.002 (−0.010 to 0.006)	.32 .67	PRS: −0.002 (−0.010 to 0.006) wPRS: −0.011 (−0.036 to 0.014)	.67 .38
	Urinary incontinence	PRS: −0.001 (−0.011 to 0.010) wPRS: 0.011 (−0.054 to 0.075)	.88 .73	PRS: 0.011 (−0.053 to 0.076) wPRS: −0.102 (−0.296 to 0.091)	.73 .30
	Decreased stream	PRS: −0.001 (−0.006 to 0.005) wPRS: 0.004 (−0.030 to 0.039)	.76 .80	PRS: 0.005 (−0.030 to 0.039) wPRS: −0.059 (−0.162 to 0.045)	.80 .26
	Dysuria	PRS: −0.002 (−0.011 to 0.007) wPRS: −0.021 (−0.075 to 0.034)	.66 .46	PRS: −0.021 (−0.075 to 0.034) wPRS: −0.017 (−0.181 to 0.147)	.46 .84
	Gastrointestinal incontinence	PRS: −0.003 (−0.011 to 0.004) wPRS: −0.026 (−0.073 to 0.022)	.38 .29	PRS: −0.026 (−0.073 to 0.022) wPRS: −0.105 (−0.248 to 0.039)	.29 .15
	Diarrhea	PRS: −0.004 (−0.010 to 0.002) wPRS: −0.015 (−0.054 to 0.024)	.19 .46	PRS: −0.015 (−0.054 to 0.024) wPRS: −0.089 (−0.207 to 0.029)	.46 .14
	Gastrointestinal urgency	PRS: −0.004 (−0.016 to 0.008) wPRS: −0.024 (−0.101 to 0.052)	.48 .53	PRS: −0.024 (−0.101 to 0.052) wPRS: −0.059 (−0.288 to 0.170)	.53 .62
	Tenesmus	PRS: −0.005 (−0.015 to 0.004) wPRS: −0.023 (−0.085 to 0.039)	.28 .47	PRS: −0.023 (−0.085 to 0.039) wPRS: −0.110 (−0.297 to 0.077)	.47 .23
	Gastrointestinal pain	PRS: 0.008 (0.000 to 0.015) wPRS: 0.053 (0.006 to 0.100)	.05 .03	PRS: 0.053 (0.006 to 0.010) wPRS: 0.060 (−0.083 to 0.203)	.03 .41
	Rectal bleeding	PRS: −0.004 (−0.014 to 0.006) wPRS: −0.052 (−0.115 to 0.011)	.41 .10	PRS: −0.054 (−0.115 to 0.010) **wPRS: −0.295 (−0.483 to −0.107)**	.10 **.002**
	Constipation	PRS: 0.000 (−0.004 to 0.004) wPRS: −0.009 (−0.035 to 0.016)	.95 .48	PRS: −0.009 (−0.035 to 0.016) wPRS: −0.032 (−0.110 to 0.045)	.48 .42
Lung	Cough	PRS: −0.001 (−0.013 to 0.010) wPRS: −0.011 (−0.085 to 0.063)	.81 .77	PRS: −0.059 (−0.280 to 0.161) wPRS: −0.130 (−0.349 to 0.090)	.60 .25
	Dyspnea	PRS: −0.007 (−0.022 to 0.008) wPRS: −0.039 (−0.134 to 0.057)	.39 .43	PRS: −0.251 (−0.536 to 0.034) wPRS: −0.063 (−0.348 to 0.223)	.09 .67
	Pneumonitis	PRS: 0.001 (−0.009 to 0.012) wPRS: 0.032 (−0.033 to 0.098)	.79 .33	PRS: 0.143 (−0.053 to 0.338) wPRS: 0.082 (−0.113 to 0.278)	.15 .41
	Dysphagia	PRS: −0.003 (−0.011 to 0.006) wPRS: −0.024 (−0.080 to 0.032)	.58 .39	PRS: −0.040 (−0.208 to 0.129) wPRS: −0.044 (−0.212 to 0.124)	.65 .61
	Esophagitis	PRS: −0.006 (−0.016 to 0.004) wPRS: −0.001 (−0.062 to 0.060)	.23 .98	PRS: 0.062 (−0.122 to 0.245) wPRS: 0.068 (−0.115 to 0.251)	.51 .47
Breast	Erythema	PRS: −0.003 (−0.010 to 0.004) wPRS: 0.013 (−0.030 to 0.056)	.47 .55	PRS: 0.038 (−0.095 to 0.170) wPRS: −0.023 (−0.155 to 0.109)	.58 .73
	Ulceration	PRS: −0.002 (−0.007 to 0.004) wPRS: −0.020 (−0.052 to 0.013)	.53 .24	PRS: −0.041 (−0.141 to 0.060) wPRS: −0.085 (−0.185 to 0.015)	.43 .10

Abbreviations: CI = confidence interval; IMRT = Intensity Modulated Radiation Therapy; PRS = polygenic risk score; wPRS = weighted polygenic risk score.

a The multivariable analysis included the following adjustment variables: prostate (age at radiotherapy, diabetes, prior surgery, hormone therapy, prescription dose [converted to biologically effective dose]); lung (sex, age at radiotherapy, prior or current smoker, radiotherapy technique [3-dimensional conformal, arc, IMRT, tomotherapy, stereotactic radiotherapy], fev1, v20 lungs, v35 esophagus, prescription dose [converted to biologically effective dose], diagnosis of chronic obstructive pulmonary disease); and breast (age at radiotherapy, prior or current smoker, history of cardiovasculature disease, body mass index, breast volume, diagnosis of diabetes, postoperative breast infection, received breast boost). Results significant after Bonferroni corrections are shown in bold.

Multivariable results for the individual late toxicity endpoints are included in Table 3. For patients with prostate cancer, individuals in the top 10th percentile for weighted polygenic risk score had a statistically significantly lower risk of hematuria (coefficient = −0.06, 95% CI = −0.11 to −0.02; *P* = .01; Bonferroni correction for 5 late endpoints, statistically significance level *P* = .01). For patients with lung cancer, individuals in the top 10th percentile of polygenic risk score showed a statistically significant association with increased risk of esophagitis (coefficient = 0.18, 95% CI = 0.05 to 0.32; *P* = .01; Bonferroni correction for 5 late endpoints, statistically significance level *P* = .01). There were no late toxicities statistically significant for the patients with breast cancer after Bonferroni correction (Bonferroni correction for 7 late endpoints, statistically significance level *P* = .007).

**Table 3.** Multivariable analysis for individual late toxicity endpoints with polygenic risk score and weighted polygenic risk score as continuous variables and dichotomized at the 90th percentile in the REQUITE cohort^a^

Toxicity		Continuous		90th percentile	
		Beta (95% CI)	*P*	Beta (95% CI)	*P*
Prostate	Proctitis	PRS: −0.001 (−0.010 to 0.007) wPRS: 0.002 (−0.051 to 0.055)	.77 .95	PRS: 0.002 (−0.051 to 0.055) wPRS: 0.094 (−0.254 to 0.066)	.95 .25
	Rectal bleeding	PRS: 0.003 (−0.001 to 0.008) wPRS: 0.009 (−0.015 to 0.032)	.12 .46	PRS: 0.009 (−0.015 to 0.032) wPRS: 0.024 (−0.046 to 0.094)	.46 .51
	Hematuria	PRS: 0.000 (−0.003 to 0.002) wPRS: −0.006 (−0.021 to 0.009)	.88 .44	PRS: −0.006 (−0.022 to 0.010) **wPRS: −0.061 (−0.106 to −0.016)**	.44 **.01**
	Urinary frequency	PRS: 0.001 (−0.007 to 0.009) wPRS: 0.006 (−0.043 to 0.055)	.79 .80	PRS: 0.006 (−0.043 to 0.055) wPRS: 0.024 (−0.126 to 0.175)	.82 .75
	Urinary retention	PRS: 0.003 (−0.003 to 0.009) wPRS: 0.028 (−0.004 to 0.060)	.18 .08	PRS: 0.028 (−0.004 to 0.060) wPRS: −0.016 (−0.112 to 0.082)	.08 .76
Lung	Cough	PRS: 0.007 (−0.005 to 0.019) wPRS: 0.023 (−0.054 to 0.100)	.26 .55	PRS: 0.156 (−0.074 to 0.386) wPRS: 0.236 (0.007 to 0.464)	.19 .04
	Dyspnea	PRS: −0.002 (−0.019 to 0.014) wPRS: −0.022 (−0.125 to 0.081)	.77 .68	PRS: −0.022 (−0.125 to 0.081) wPRS: 0.066 (−0.241 to 0.373)	.68 .67
	Pneumonitis	PRS: 0.007 (−0.005 to 0.019) wPRS: 0.054 (−0.024 to 0.132)	.27 .18	PRS: 0.038 (−0.196 to 0.272) wPRS: 0.114 (−0.119 to 0.347)	.75 .34
	Dysphagia	PRS: 0.006 (−0.003 to 0.015) wPRS: 0.027 (−0.027 to 0.081)	.18 .33	PRS: 0.172 (0.011 to 0.333) wPRS: 0.106 (−0.055 to 0.267)	.04 .20
	Esophagitis	PRS: 0.001 (−0.006 to 0.009) wPRS: 0.002 (−0.043 to 0.046)	.70 .95	**PRS: 0.184 (0.051 to 0.317)** wPRS: 0.069 (−0.065 to 0.203)	**.01** .31
Breast	Telangiectasia	PRS: 0.000 (−0.002 to 0.002) wPRS: −0.002 (−0.015 to 0.012)	.96 .82	PRS: −0.014 (−0.057 to 0.029) wPRS: −0.012 (−0.055 to 0.031)	.53 .59
	Telangiectasia, tumor bed	PRS: −0.001 (−0.003 to 0.002) wPRS: −0.010 (−0.024 to 0.004)	.59 .17	PRS: −0.032 (−0.075 to 0.011) wPRS: 0.007 (−0.036 to 0.050)	.14 .74
	Edema	PRS: −0.001 (−0.006 to 0.003) wPRS: 0.000 (−0.026 to 0.025)	.48 .98	PRS: −0.010 (−0.089 to 0.069) wPRS: −0.021 (−0.101 to 0.058)	.80 .60
	Induration	PRS: 0.002 (−0.002 to 0.007) wPRS: −0.007 (−0.033 to 0.019)	.26 .62	PRS: 0.053 (−0.028 to 0.133) wPRS: −0.024 (−0.104 to 0.057)	.20 .56
	Induration, tumor bed	PRS: −0.001 (−0.006 to 0.004) wPRS: −0.012 (−0.044 to 0.020)	.72 .47	PRS: 0.011 (−0.087 to 0.109) wPRS: −0.087 (−0.185 to 0.012)	.82 .08
	Pigmentation	PRS: 0.004 (0.00 to 0.008) wPRS: 0.018 (−0.007 to 0.043)	.08 .15	PRS: 0.077 (0.000 to 0.155) wPRS: 0.038 (−0.040 to 0.115)	.05 .34
	Atrophy	PRS: 0.001 (−0.005 to 0.007) wPRS: −0.007 (−0.043 to 0.028)	.76 .68	PRS: −0.005 (−0.113 to 0.103) wPRS: 0.006 (−0.103 to 0.115)	.93 .91

Abbreviations: CI = confidence interval; IMRT = Intensity Modulated Radiation Therapy; PRS = polygenic risk score; wPRS = weighted polygenic risk score.

a The multivariable analysis included the following adjustment variables: prostate (age at radiotherapy, diabetes, prior surgery, hormone therapy, prescription dose [converted to biologically effective dose]); lung (sex, age at radiotherapy, prior or current smoker, radiotherapy technique [3-dimensional conformal, arc, IMRT, tomotherapy, stereotactic radiotherapy]), fev1, v20 lungs, v35 esophagus, prescription dose [converted to biologically effective dose]), diagnosis of chronic obstructive pulmonary disease; breast (age at radiotherapy, prior or current smoker, history of cardiovasculature disease, body mass index, breast volume, diagnosis of diabetes, postoperative breast infection, received breast boost). Results significant after Bonferroni corrections are shown in bold.

Validation results are presented in Table S5. STAT-acute, STAT-late, and individual endpoints were analyzed for each cohort. Multivariable models used the same adjustment variables as described in the primary analysis. No statistically significant results were identified after Bonferroni corrections for the number of endpoints tested. Univariable and multivariable models of STAT-acute and STAT-late by RA diagnosis phenotype using the same adjustment variables as described in the primary analysis did not identify statistically significant results (see Table S5).

## Discussion

In this work, we have calculated a polygenic risk score for RA in patients treated with radiotherapy for prostate, breast, or lung cancer. No association between the polygenic risk score (or weighted polygenic risk score) was found for STAT-acute or STAT-late in the primary dataset or in any of the validation datasets. Analyzing individual endpoints showed patients in the top 10th percentile of polygenic risk score, treated with radiotherapy for lung cancer, had an increased risk of late esophagitis (*P* = .01). For patients with prostate cancer, those in the top 10th percentile of weighted polygenic risk score had an increased risk of acute rectal bleeding and increased risk of late hematuria. However, none of these individual toxicity endpoints were validated in the independent cohorts. Overall, the analysis suggests that patients with a high polygenic risk score for RA do not experience increased toxicities from receiving radiotherapy as part of their cancer treatment. These results provide evidence that treatment planning techniques or organ at risk (OAR) dose constraints do not need be modified for such patients.

The RA phenotype is complex, and there has been conflicting evidence published suggesting patients with RA are at higher risk of radiation-induced toxicity.^1^ The use of the polygenic risk score for RA in this work allows the genetic component to be analyzed as the primary driver behind increased toxicities. This hypothesis was of interest as there exists overlaps of genes with RA and DNA repair pathways. However, as this work identified no statistically significance, it may be other aspects of the RA phenotype that result in these observer clinical profiles. For example, RA itself may initiate a cascade of inflammation. This increased inflammatory state of RA patients may then predispose an individual to increased risk of radiation-induced toxicities. These interlinked aspects need to be considered in future studies, with measures of an individuals’ inflammatory state and germline DNA for the calculation of their genetic risk of RA needed to separate these influences.

A polygenic risk score is becoming a standard approach for calculating the relative and absolute risk for an individual in developing a complex disease affected by the interaction of numerous single-nucleotide variation (SNV).^30^ These scores only provide an estimated risk but do not predict when the disease will manifest. They also combine the contribution of numerous SNPs into a single value. However, in the context of the approach in this paper, it may be a smaller subset of SNPs, common between DNA repair and RA, which drives the increased toxicity (for example, those in *ATM* and *RAD51B*^2^^,^^7^). This level of granularity is not captured within the polygenic risk score, where an individual’s high genetic risk for RA may be through contribution of high expression of SNPs, which do not increase toxicity.

GWAS analysis for RA and other complex disease are increasing in size and power. A recent GWAS for RA analyzed the data from 276 020 individuals, identifying more than 2000 risk loci.^31^ With this number of SNPs identified, care is needed in defining the polygenic risk score to minimize the impact of many SNPs with a low effect size.^22^ An emerging approach to consider is to partition the polygenic risk score.^32^^,^^33^ This approach clusters the SNPs identified from the GWAS with biological process-specific mechanisms. This has been successfully performed for type 2 diabetes with clusters defined around SNPs involved in biological processes associated with B cell, proinsulin, obesity, lipodystrophy, and lipids.^34^ These partitions were then associated with cardiovascular endpoints, with different partitions showing a stronger association with different endpoints than the combined polygenic risk score. This approach could be readily adopted in future work in understanding associations with normal tissue toxicities. Partitioning the RA GWAS into biological mechanisms around potential pathways associated with toxicity (eg, inflammation, DNA repair) may better power future analyses.

RA is a complex disease that affects patients through multiple mechanisms leading to a common clinical phenotype.^35^ Mechanisms work through environmental and genetic factors and include cell matrix destruction, inflammation (local and systemic), and a breakdown of immune tolerance. The multifaceted nature of RA brings a level of noise into the analysis. The GWAS^2^ did not discriminate between mechanisms, only analyzing against the clinical phenotype, therefore the identified SNPs will capture all mechanisms. It is likely that different mechanisms involved in the development of RA will impact patient toxicity outcomes differentially. These considerations may help explain the conflicting reports in the literature and the lack of clear clinical guidance for the management of patients with RA undergoing radiotherapy. There may also be influences from polypharmacy; patients with managed RA may experience increased or decreased radiotherapy-related toxicity risk from interactions with ongoing medications.^36^^,^^37^ Therefore, radiation oncologists should have a discussion with rheumatologists and general practitioners regarding the importance of continuing disease-modifying medication during radiotherapy. These considerations should be further explored in datasets where details on medications are available.

Preselected adjustment variables were controlled in multivariable models. These were selected based on established knowledge of drivers of normal tissue toxicities. There may be further variables unaccounted that would improve model performance. Additionally, toxicity data were only available up to 2 years postradiotherapy; some radiation-induced toxicity can develop even later and further analysis should be considered to look at longer-term presentation of toxicities. A further aspect worth considering in future studies is to better account for the dosimetric drivers of toxicity. Numerous dosimetric studies have now highlighted dose-sensitive subregions of organs at risk where excess dose is more strongly associated with toxicity and morbidity than whole organ doses. For example, work in patients treated with radiotherapy for prostate cancer have identified different spatial dose patterns across the surface of the rectum^38^ and bladder^39^^,^^40^ associated with different toxicity endpoints. Similarly, in lung cancer patients, the base of the heart has been identified as more dose sensitive.^41^^,^^42^ By better combining spatial dose modeling alongside genetic drivers of toxicity, a more detailed understanding of dosimetric and genetic drivers of toxicity will emerge. In this work, we remained focused on toxicities affecting normal tissues within the radiation field. However, a further common acute radiation-induced toxicity experienced is fatigue, because of potential interactions of the radiation and the immune system. Fatigue can also be associated with an increased inflammatory state, and there may be interactions with RA. A follow-up study should investigate if patients with a high genetic risk of RA experience worsens fatigue postradiotherapy.

In conclusion, patients with a high genetic risk of RA do not appear to have an increased risk of normal tissue toxicity after receiving radiotherapy. These results suggest that radiotherapy planning and delivery does not need to be modified for these patients.

## Supplementary Material

djae349_Supplementary_Data

## Data Availability

Data can be made available through request to the REQUITE trial management committee.

## References

[1] Liebenberg N , Mcwilliam A, Kerns SL, Marshall DC, West CM. The association between rheumatoid arthritis and risk of radiotherapy toxicity: a systematic review. BMJ Oncol. 2024;3:e000407. 10.1136/bmjonc-2024-000407 PMC11256021 39524982

[2] Okada Y , Wu D, Trynka G, et al; GARNET Consortium. Genetics of rheumatoid arthritis contributes to biology and drug discovery. Nature. 2014;506:376-381. 24390342 10.1038/nature12873 PMC3944098

[3] McAllister K , Yarwood A, Bowes J, et al Identification of BACH2 and RAD51B as rheumatoid arthritis susceptibility loci in a meta-analysis of genome-wide data. Arthritis Rheumatol. 2013;65:3058-3062. 10.1002/art.38183 PMC4034583 24022229

[4] Jiang L , Yin J, Ye L, et al Novel risk loci for rheumatoid arthritis in Han Chinese and congruence with risk variants in Europeans. Arthritis Rheumatol. 2014;66:1121-1132. 24782177 10.1002/art.38353

[5] Chistiakov DA , Voronova NV, Chistiakov PA. Genetic variations in DNA repair genes, radiosensitivity to cancer and susceptibility to acute tissue reactions in radiotherapy-treated cancer patients. Acta Oncol. 2008;47:809-824. 18568480 10.1080/02841860801885969

[6] Gatti RA. The inherited basis of human radiosensitivity. Acta Oncol. 2001;40:702-711. 11765064 10.1080/02841860152619115

[7] Vignard J , Mirey G, Salles B. Ionizing-radiation induced DNA double-strand breaks: a direct and indirect lighting up. Radiother Oncol. 2013;108:362-369. 23849169 10.1016/j.radonc.2013.06.013

[8] Santivasi WL , Xia F. Ionizing radiation-induced DNA damage, response, and repair. Antioxid Redox Signal. 2014;21:251-259. 24180216 10.1089/ars.2013.5668

[9] Fodil N , Langlais D, Gros P. Primary immunodeficiencies and inflammatory disease: a growing genetic intersection. Trends Immunol. 2016;37:126-140. 26791050 10.1016/j.it.2015.12.006 PMC4738049

[10] Chun HH , Gatti RA. Ataxia-telangiectasia, an evolving phenotype. DNA Repair (Amst). 2004;3:1187-1196. 15279807 10.1016/j.dnarep.2004.04.010

[11] Calabresi E , Petrelli F, Bonifacio AF, Puxeddu I, Alunno A. One year in review 2018: pathogenesis of rheumatoid arthritis. Clin Exp Rheumatol. 2018;36:175-184. 29716677

[12] Kim JH , Jenrow KA, Brown SL. Mechanisms of radiation-induced normal tissue toxicity and implications for future clinical trials. Radiat Oncol J. 2014;32:103-115. 25324981 10.3857/roj.2014.32.3.103 PMC4194292

[13] Najafi M , Motevaseli E, Shirazi A, et al Mechanisms of inflammatory responses to radiation and normal tissues toxicity: clinical implications. Int J Radiat Biol. 2018;94:335-356. 29504497 10.1080/09553002.2018.1440092

[14] Felefly T , Mazeron R, Huertas A, et al Pelvic radiotherapy in the setting of rheumatoid arthritis: refining the paradigm. Cancer Radiother. 2017;21:109-113. 28363728 10.1016/j.canrad.2016.09.020

[15] Dong Y , Li T, Churilla TM, et al Impact of rheumatoid arthritis on radiation-related toxicity and cosmesis in breast cancer patients: a contemporary matched-pair analysis. Breast Cancer Res Treat. 2017;166:787-791. 28825145 10.1007/s10549-017-4438-7

[16] Chen K-H , Zhu X-D, Li L, et al Comparison of the efficacy between concurrent chemoradiotherapy with or without adjuvant chemotherapy and intensity-modulated radiotherapy alone for stage II nasopharyngeal carcinoma. Oncotarget. 2016;7:69041-69050. 27634892 10.18632/oncotarget.11978 PMC5356610

[17] Lin D , Lehrer EJ, Rosenberg J, Trifiletti DM, Zaorsky NG. Toxicity after radiotherapy in patients with historically accepted contraindications to treatment (CONTRAD): an international systematic review and meta-analysis. Radiother Oncol. 2019;135:147-152. 31015161 10.1016/j.radonc.2019.03.006

[18] Lin A , Abu-Isa E, Griffith KA, Ben-Josef E. Toxicity of radiotherapy in patients with collagen vascular disease. Cancer. 2008;113:648-653. 18506734 10.1002/cncr.23591

[19] Diao K , Chen Y-H, Catalano PJ, et al Radiation toxicity in patients with collagen vascular disease and intrathoracic malignancy treated with modern radiation techniques. Radiother Oncol. 2017;125:301-309. 29102264 10.1016/j.radonc.2017.10.002

[20] Kerns SL , de Ruysscher D, Andreassen CN, et al STROGAR—STrengthening the Reporting Of Genetic Association studies in Radiogenomics. Radiother Oncol. 2014;110:182-188. 23993398 10.1016/j.radonc.2013.07.011 PMC4786020

[21] West C , Azria D, Chang-Claude J, et al The REQUITE project: validating predictive models and biomarkers of radiotherapy toxicity to reduce side-effects and improve quality of life in cancer survivors. Clin Oncol (R Coll Radiol). 2014;26:739-742. 25267305 10.1016/j.clon.2014.09.008

[22] Seibold P , Webb A, Aguado-Barrera ME, et al; REQUITE Consortium. REQUITE: a prospective multicentre cohort study of patients undergoing radiotherapy for breast, lung or prostate cancer. Radiother Oncol. 2019;138:59-67. 31146072 10.1016/j.radonc.2019.04.034

[23] Dearnaley DP , Sydes MR, Graham JD, et al; RT01 Collaborators. Escalated-dose versus standard-dose conformal radiotherapy in prostate cancer: first results from the MRC RT01 randomised controlled trial. Lancet Oncol. 2007;8:475-487. 17482880 10.1016/S1470-2045(07)70143-2

[24] Dearnaley D , Syndikus I, Mossop H, et al; CHHiP Investigators. Conventional versus hypofractionated high-dose intensity-modulated radiotherapy for prostate cancer: 5-year outcomes of the randomised, non-inferiority, phase 3 CHHiP trial. Lancet Oncol. 2016;17:1047-1060. 27339115 10.1016/S1470-2045(16)30102-4 PMC4961874

[25] West C , Rosenstein BS, Alsner J, et al; EQUAL-ESTRO. Establishment of a radiogenomics consortium. Int J Radiat Oncol Biol Phys. 2010;76:1295-1296. 20338472 10.1016/j.ijrobp.2009.12.017

[26] Mukesh MB , Barnett GC, Wilkinson JS, et al Randomized controlled trial of intensity-modulated radiotherapy for early breast cancer: 5-year results confirm superior overall cosmesis. J Clin Oncol Off J Am Soc Clin Oncol. 2013;31:4488-4495. 10.1200/JCO.2013.49.7842 24043742

[27] Fachal L , Gómez-Caamaño A, Barnett GC, et al A three-stage genome-wide association study identifies a susceptibility locus for late radiotherapy toxicity at 2q24.1. Nat Genet. 2014;46:891-894. 24974847 10.1038/ng.3020

[28] Aguado-Barrera ME , Martínez-Calvo L, Fernández-Tajes J, et al Validation of polymorphisms associated with the risk of radiation-induced oesophagitis in an independent cohort of non-small-cell lung cancer patients. Cancers. 2021;13:1447-2021. 33810047 10.3390/cancers13061447 PMC8004670

[29] Barnett GC , West CML, Coles CE, et al Standardized Total Average Toxicity score: a scale- and grade-independent measure of late radiotherapy toxicity to facilitate pooling of data from different studies. Int J Radiat Oncol Biol Phys. 2012;82:1065-1074. 21605943 10.1016/j.ijrobp.2011.03.015

[30] Lewis CM , Vassos E. Polygenic risk scores: from research tools to clinical instruments. Genome Med. 2020;12:44. 32423490 10.1186/s13073-020-00742-5 PMC7236300

[31] Ishigaki K , Sakaue S, Terao C, et al; BioBank Japan Project. Multi-ancestry genome-wide association analyses identify novel genetic mechanisms in rheumatoid arthritis. Nat Genet. 2022;54:1640-1651. 36333501 10.1038/s41588-022-01213-w PMC10165422

[32] Tan VY , Fevotte C. Automatic relevance determination in nonnegative matrix factorization with the beta-divergence. IEEE Trans Pattern Anal Mach Intell. 2013;35:1592-1605. 23681989 10.1109/TPAMI.2012.240

[33] Kim J , Mouw KW, Polak P, et al Somatic ERCC2 mutations are associated with a distinct genomic signature in urothelial tumors. Nat Genet. 2016;48:600-606. 27111033 10.1038/ng.3557 PMC4936490

[34] Udler MS , Kim J, von Grotthuss M, et al; on behalf of METASTROKE and the ISGC. Type 2 diabetes genetic loci informed by multi-trait associations point to disease mechanisms and subtypes: a soft clustering analysis. PLoS Med. 2018;15:e1002654. 30240442 10.1371/journal.pmed.1002654 PMC6150463

[35] Gravallese EM , Firestein GS. Rheumatoid arthritis—common origins, divergent mechanisms. N Engl J Med. 2023;388:529-542. 36780677 10.1056/NEJMra2103726

[36] Morris MM , Powell SN. Irradiation in the setting of collagen vascular disease: acute and late complications. J Clin Oncol. 1997;15:2728-2735. 9215847 10.1200/JCO.1997.15.7.2728

[37] Yamamoto K , Nagao S, Suzuki K, et al Pelvic fractures after definitive and postoperative radiotherapy for cervical cancer: a retrospective analysis of risk factors. Gynecol Oncol. 2017;147:585-588. 29055558 10.1016/j.ygyno.2017.09.035

[38] Shelley LEA , Sutcliffe MPF, Thomas SJ, et al Associations between voxel-level accumulated dose and rectal toxicity in prostate radiotherapy. Phys Imaging Radiat Oncol. 2020;14:87-94. 32582869 10.1016/j.phro.2020.05.006 PMC7301619

[39] Heemsbergen WD , Al-Mamgani A, Witte MG, van Herk M, Pos FJ, Lebesque JV. Urinary obstruction in prostate cancer patients from the Dutch trial (68 Gy vs. 78 Gy): relationships with local dose, acute effects, and baseline characteristics. Int J Radiat Oncol Biol Phys. 2010;78:19-25. 20056354 10.1016/j.ijrobp.2009.07.1680

[40] Onjukka E , Fiorino C, Cicchetti A, et al Patterns in ano-rectal dose maps and the risk of late toxicity after prostate IMRT. Acta Oncol. 2019;58:1757-1764. 31298076 10.1080/0284186X.2019.1635267

[41] Craddock M , Nestle U, Koenig J, et al Cardiac function modifies the impact of heart base dose on survival: a voxel-wise analysis of patients with lung cancer from the PET-plan trial. J Thorac Oncol. 2023;18:57-66. 36130693 10.1016/j.jtho.2022.09.004

[42] McWilliam A , Abravan A, Banfill K, Faivre-Finn C, van Herk M. Demystifying the results of RTOG 0617: identification of dose sensitive cardiac subregions associated with overall survival. J Thorac Oncol. 2023;18:599-607. 36738929 10.1016/j.jtho.2023.01.085

