# Supplementary material for: Association of radiation-induced normal tissue toxicity with a high genetic risk for rheumatoid arthritis
Source: J Natl Cancer Inst. 2025 Jan 6;117(5):1018–26. doi: 10.1093/jnci/djae349 (PMC12058264; doi:10.1093/jnci/djae349)
Supplement: djae349_Supplementary_Data [file djae349_supplementary_data.pdf]

Supplementary Material
Supplementary tables 1 – Toxicity definitions
The toxicity definitions used in each cohort are included here. For REQUITE the validated patient reported outcome questionnaires are described by Seibold et al [1].

Prostate cohorts
REQUITE

REQUITE	Urinary frequency	0 = Normal<br>1 = Up to twice more often than normal<br>2 = Over twice as often as normal
	Nocturia	0 = None<br>1 = Once per night<br>2 = 2 to 3 times per night<br>3 = 4 to 6 times per night<br>4 = 7 or more times per night
	Urinary urgency	0 = None at all<br>1 = Monthly or less<br>2 = Weekly<br>3 = Daily<br>4 = Constantly
	Haematuria	0 = None<br>1 = Gross bleeding<br>2 = Gross bleeding with treatment needed
	Urinary incontinence	0 = None<br>1 = Monthly<br>2 = Weekly<br>3 = Daily<br>4 = Constantly
	Decreased urinary stream	0 = Normal<br>1 = Hesitancy or dribbling<br>2 = Requiring medication/catheter<br>3 = Daily catheterization needed
	Dysuria	0 = None at all<br>1 = Minimal<br>2 = Tolerable with painkillers (but does not interfere with activities)<br>3 = Intense, requires painkillers (interferes with activities)<br>4 = Excruciating (interferes with all activities)
	Rectal bleeding	0 = None<br>1 = Gross bleeding<br>2 = Gross bleeding with treatment needed
	Gastro-intestinal incontinence	0 = None<br>1 = Monthly<br>2 = Weekly<br>3 = Daily<br>4 = Constantly
	Diarrhoea	0 = None or same occurrence as usual<br>1 = Increase up to 4 times daily<br>2 = Increase of 4 to 6 times daily<br>3 = Increase of 7 or more times daily
	Gastro-intestinal urgency	0 = None at all<br>1 = Monthly or less<br>2 = Weekly<br>3 = Daily<br>4 = Constantly
	Tenesmus	0 = None at all<br>1 = Monthly or less<br>2 = Weekly<br>3 = Daily

		4 = Constantly
	Gastro-intestinal pain	0 = None at all<br>1 = Minimal<br>2 = Tolerable with painkillers (but does not interfere with activities)<br>3 = Intense, requires painkillers (interferes with activities)<br>4 = Excruciating (interferes with all activities)
	Proctitis	0 = None at all<br>1 = Minimal<br>2 = Tolerable with painkillers (but does not interfere with activities)<br>3 = Intense, requires painkillers (interferes with activities)<br>4 = Excruciating (interferes with all activities)
	Rectal mucus loss	0 = None<br>1 = Rarely<br>2 = Sometimes<br>3 = Often<br>4 = Always
	Constipation	0 = None, bowel movement at least 4 times per week<br>1 = Bowel movement 3 to 4 times per week<br>2 = Bowel movement 2 times per week<br>3 = Bowel movement once per week<br>4 = Bowel movement less than once per week

Prostate validation cohorts from the Radiogenomic Consortium

	**STATacute**	**STATlate-Obs**
**CHHiP (N=1,985); RT01 (N=252)**		RMH frequency<br>RMH rectal bleeding<br>RTOG proctitis<br>RTOG hematuria<br>RTOG retention
**NIRS (N=712)**		RTOG frequency<br>RTOG retention<br>RTOG hematuria<br>RTOG proctitis<br>rectal bleeding N/A
**RADIOGEN_Prostate (N=655)**		CTCAEv3:<br>GI bleeding<br>proctitis<br>urinary frequency/urgency<br>cystitis<br>urinary retention
**MSSM (N=650)**	IPSS:<br>urinary frequency, weak stream, incomplete emptying, intermittency, urgency, straining, nocturia	Combination of PRO and Obs measures; included in both analyses:<br>IPSS urinary frequency<br>IPSS urinary weak stream<br>hematuria yes/no (‘yes’ graded 2)<br>RTOG rectal bleeding/proctitis
**UGhent (N=315)**		In-house scale:<br>rectal bleeding<br>rectitis<br>frequency<br>hematuria<br>retention N/A
**CCI-BT (N=274)**		Combination of PRO and Obs measures; included in both analyses:<br>IPSS urinary frequency<br>IPSS urinary weak stream

		RTOG rectal bleeding/proctitis<br>hematuria N/A
**RT01 (N=252)**		RMH frequency<br>RMH rectal bleeding<br>RTOG proctitis<br>RTOG hematuria<br>RTOG retention
**NTMC (N=252)**	IPSS:<br>urinary frequency, weak stream, incomplete emptying, intermittency, urgency, straining, nocturia	Combination of PRO and Obs measures; included in both analyses:<br>IPSS urinary frequency<br>IPSS urinary weak stream<br>RTOG rectal bleeding/proctitis<br>hematuria N/A
**URWCI-MCW (N=247)**		CTCAEv4:<br>GI bleeding<br>proctitis<br>urinary frequency/urgency<br>cystitis<br>urinary retention
**CCI-EBRT (N=151)**		CTCAEv3:<br>GI bleeding<br>proctitis<br>urinary frequency/urgency<br>cystitis<br>urinary retention

Lung cohorts

REQUITE

REQUITE	Cough	0 = None<br>1 = Mild symptoms; non-prescription intervention indicated<br>2 = Moderate symptoms; medical intervention indicated; limiting instrumental activities of daily living (ADL)<br>3 = Severe symptoms; limiting self-care ADL
	Dyspnoea	0 = None<br>1 = Shortness of breath with moderate exertion<br>2 = Shortness of breath with minimal exertion; limiting instrumental ADL<br>3 = Shortness of breath at rest; limiting self-care ADL<br>4 = Life-threatening consequences; urgent intervention indicated
	Pneumonitis	0 = None<br>1 = Asymptomatic; clinical or diagnostic observations only; intervention not indicated<br>2 = Symptomatic; medical intervention indicated; limiting instrumental ADL<br>3 = Severe symptoms; limiting self-care ADL; oxygen indicated<br>4 = Life-threatening respiratory compromise; urgent intervention indicated (e.g., tracheotomy or intubation)
	Oesophagitis	0 = None<br>1 = Asymptomatic; clinical or diagnostic observations only; intervention not indicated<br>2 = Symptomatic; altered GI function<br>3 = Severely altered GI function; tube feeding; hospitalization indicated; elective operative intervention indicated<br>4 = Life-threatening consequences; urgent operative intervention indicated

	Dysphagia	0 = None<br>1 = Symptomatic, able to eat regular diet<br>2 = Symptomatic and altered eating/swallowing<br>3 = Severely altered eating/swallowing; tube feeding or total parenteral nutrition (TPN) or hospitalization indicated<br>4 = Life-threatening consequences; urgent operative intervention indicated

Validation cohort available from the RADIOGEN-Lung cohort.

Toxicity endopoint and scale	Grade Description
Cough (CTCAE v.4.0)	0 = None<br>1 = Mild symptoms; nonprescription intervention indicated<br>2 = Moderate symptoms, medical intervention indicated; limiting instrumental ADL<br>3 = Severe symptoms; limiting self care ADL
Dyspnea (CTCAE v.4.0)	0 = None<br>1 = Shortness of breath with moderate exertion<br>2 = Shortness of breath with minimal exertion; limiting instrumental ADL<br>3 = Shortness of breath at rest; limiting self care ADL<br>4 = Life-threatening consequences; urgent intervention indicated<br>5 = Death
Pneumonitis (CTCAE v.4.0)	0 = None<br>1 = Asymptomatic; clinical or diagnostic observations only; intervention not indicated<br>2 = Symptomatic; medical intervention indicated; limiting instrumental ADL<br>3 = Severe symptoms; limiting self care ADL; oxygen indicated<br>4 = Life-threatening respiratory compromise; urgent intervention indicated (e.g., tracheotomy or intubation)<br>5 = Death
Esophagitis (CTCAE v.4.0)	0 = None<br>1 = Asymptomatic; clinical or diagnostic observations only; intervention not indicated<br>2 = Symptomatic; altered eating/swallowing; oral supplements indicated<br>3 = Severely altered eating/swallowing; tube feeding, TPN or hospitalization indicated<br>4 = Life-threatening consequences; urgent operative intervention indicated<br>5 = Death
Dysphagia (CTCAE v.4.0)	0 = None<br>1 = Symptomatic, able to eat regular diet<br>2 = Symptomatic and altered eating/swallowing<br>3 = Severely altered eating/swallowing; tube feeding or TPN or hospitalization indicated<br>4 = Life-threatening consequences; urgent intervention indicated<br>5 = Death

Breast cohorts

REQUITE

REQUITE	Erythema	1 = Faint erythema or dry desquamation.<br>2 = Moderate to brisk erythema; patchy moist desquamation, mostly confined to skin folds and creases; moderate oedema<br>3 = Moist desquamation in areas other than skin folds and creases; bleeding induced by minor trauma or abrasion.<br>4 = Life-threatening consequences; skin necrosis or ulceration of full thickness dermis; spontaneous bleeding from involved site; skin graft indicated
	Ulceration	1 = Combined area of ulcers <1cm; non-blanchable erythema of intact skin with associated warmth or oedema<br>2 = Combined area of ulcers 1 – 2 cm; partial thickness skin loss involving skin or subcutaneous fat<br>3 = Combined area of ulcers >2 cm; full thickness skin loss involving damage to or necrosis of subcutaneous tissue that may extend down to fascia<br>4 = Any size ulcer with extensive destruction, tissue necrosis, or damage to muscle, bone, or supporting structures with or without full thickness skin loss
	Breast atrophy	1 = Minimal asymmetry<br>2 = Moderate asymmetry<br>3 = Asymmetry >1/3 of breast volume
	Breast oedema	1 = Localized to dependent areas, no disability or functional impairment<br>2 = Moderate localized oedema and intervention indicated; limiting instrumental ADL<br>3 = Severe localized oedema and intervention indicated; limiting self care and ADL
	Telangiectasia	1 = Telangiectasia covering <10% BSA<br>2 = Telangiectasia covering >10% BSA; associated with psychosocial impact
	Induration	1 = Mild induration, able to move skin parallel to plane (sliding) and perpendicular to skin (pinching up)<br>2 = Moderate induration, able to slide skin, unable to pinch skin; limiting instrumental ADL<br>3 = Severe induration, unable to slide or pinch skin; limiting joint movement or orifice (e.g., mouth, anus); limiting self-care ADL
	Hyper-pigmentation	1 = Hyperpigmentation covering <10% BSA; no psychosocial impact<br>2 = Hyperpigmentation covering >10% BSA; associated psychosocial impact

Validation cohort available from the Cambridge IMRT trial.

Toxicity endpoint and scale	Grade
Acute toxicity (RTOG) week 3	0 = No change over baseline<br>1 = Follicular, faint, or dull erythema/epilation/ dry/ desquamation/ decreased sweating<br>2 = Tender or bright erythema, patchy moist desquamation/moderate oedema<br>3 = Confluent, moist desquamation other than skin folds, pitting oedema<br>4 = Ulceration, haemorrhage, necrosis
**Photographic assessment** of late shrinkage and distortion (IMRT Trial and RACE)	1 = none/minimal<br>2 = mild<br>3 = marked
**Clinical assessment** of late toxicity	
Shrinkage	0 = None<br>1 = A little
Telangiectasia	

Induration	2 = Quite a bit<br>3 = Very much
Breast oedema	
**Clinical assessment** (LENT-SOM) of late toxicity	
Retraction / Atrophy	0 = Nil<br>1 = 10%-25%*<br>2 = >25%-40%<br>3 = >40%-75%<br>4 = Whole breast
Telangiectasia	0 = None<br>1 = Minimal (>1 per cm^2)<br>2 = Moderate (1-4 per cm^2)<br>3 = Severe (>4 per cm^2)
Fibrosis	0 = None<br>1 = Barely Palpable/increased density<br>2 = Definite increased density & firmness<br>3 = Marked density, retraction & fixation
Pigmentation	0 = None<br>1 = Transitory, slight<br>2 = Permanent, marked
Breast oedema	0 = None<br>1 = Asymptomatic<br>2 = Symptomatic<br>3 = Secondary dysfunction
**Patient reported** (EORTC BR23)	
Breast pain	1 = Not at all<br>2 = A little<br>3 = Quite a bit<br>4 = Very much
Breast sensation	

Supplementary methods 1 – Ethics statements for each cohort
The REQUITE study [1,2] was approved by local ethics committees and is registered at www.controlled-trials.com ISRCTN98496463. The multicentre REQUITE cohort was recruited prospectively in seven European countries and the USA between 2014 and 2016. Patient characteristics and methodology have been described in detail elsewhere [1].

Validation datasets were available from: RT01[3] (ISRCTN47772397) and CHHiP[4] (ISRCTN97182923) trials, and was approved by the Cambridge South Research Ethics Committee (05/Q0108/365). Other prostate cohorts available through data sharing agreements through the international Radiogenomics Consortium (RCG)[5]. The breast validation was available from (UKCRN1471) recruited participants enrolled in the Cambridge IMRT Trial (ISRCTN21474421)[6]. The lung validation cohort was available from RADIOGEN recruited participants treated at the Clinical University Hospital of Santiago de Compostela, Spain and was approved by the Galician Ethical Committee[7,8].

Supplementary tables 2 – Patient characteristics

Demographics and clinical characteristics of the REQUITE prostate cancer cohort.

**Characteristic**		**REQUITE N = 1494**
Age treatment	*Mean (range)*	70 (46 – 88)
Diabetes	*Yes*	194
	No	1300
Previous surgery	*Yes*	429
	No	1065
Hormone therapy	*Yes*	1080
	No	414
Prescription dose (BED)	*Mean (range)*	88.8 (28.8 – 96)
Diagnosis of RA	*Yes*	29
	No	1465

BED – biologically equivalent dose, alpha / beta = 10; RA – Rheumatoid arthritis.

Validation: Demographics and clinical characteristics of the prostate cancer cohort available for validation from the Radiogenomics Consortium.

	CHHiP	NIRS	RADIOGEN_Prostate	MSSM	UGhent	CCI-BT	RT01	NTMC	URWCI-MCW	CCI-EBRT
**Age, mean (range)**	68 (48 to 84)	68 (48 to 87)	70 (47 to 86)	64 (43 to 85)	65 (49 to 85)	67 (45 to 82)	65 (50 to 79)	67 (38 to 87)	69 (49 to 86)	64 (45 to 79)
**Ancestry**										
**African**	39 (2.2)	0	0	79 (12.5)	1 (0.3)	3 (1.1)	0	0	18 (7.4)	0
**Asian**	14 (0.8)	712 (100)	1 (0.2)	9 (1.4)	0	4 (1.5)	7 (2.8)	252 (100)	1 (0.4)	0
**Caucasian**	1,724 (97.0)	0	654 (99.8)	495 (78.2)	314 (99.7)	266 (97.4)	239 (96.8)	0	222 (91.4)	151 (100)
**Hispanic**	0	0	0	50 (7.9)	0	0	1 (0.4)	0	2 (0.8)	0
**Rheumatoid arthritis**	N/A	N/A			N/A			N/A		N/A
**Yes**			4 (0.6)	25 (3.9)		18 (6.6)	20 (8.0)		3 (1.3)	
**No**			651 (99.4)	625 (96.1)		256 (93.4)	229 (92.0)		235 (98.7)	
**Diabetes**		N/A						N/A		
**Yes**	191 (9.7)		159 (24.3)	38 (5.9)	41 (13.2)	31 (11.4)	29 (11.7)		49 (19.8)	24 (16.6)
**No**	1,776 (90.3)		496 (75.7)	612 (94.1)	270 (86.8)	241 (88.6)	220 (88.3)		198 (80.2)	121 (83.4)
**Prostatectomy**										
**Yes**	0	0	127 (19.4)	4 (0.6)	102 (32.4)	0	0	0	75 (30.9)	0
**No**	1,985 (100)	712 (100)	528 (80.6)	646 (99.4)	213 (67.6)	274 (100)	252 (100)	252 (100)	168 (69.1)	151 (100)
**Hormones**										
**Yes**	1,985 (100)	565 (79.4)	462 (70.6)	343 (52.8)	195 (63.3)	61 (22.3)	252 (100)	154 (61.4)	162 (65.8)	74 (49.7)
**No**	0	147 (20.6)	192 (29.4)	307 (47.2)	113 (36.7)	212 (77.7)	0	97 (38.6)	84 (34.2)	75 (50.3)
**RT type**										
**EBRT**	1,985 (100)	171 (24.0)	655 (100)	17 (2.6)	315 (100)	0	252 (100)	0	191 (77.3)	151 (100)
**LDR brachytherapy**	0	2 (0.3)	0	353 (54.3)	0	274 (100)	0	143 (56.7)	0	0
**EBRT + LDR brachy**	0	2 (0.3)	0	280 (43.1)	0	0	0	109 (43.3)	3 (1.2)	0
**EBRT + HDR brachy**	0	0	0	0	0	0	0	0	53 (21.5)	0
**C-ions**	0	537 (75.4)	0	0	0	0	0	0	0	0
**Total BED, mean (range)**	119 (114 to 123)	129 (52 to 158)	120 (57 to 127)	202 (52 to 320)	132 (124 to 136)	159 (80 to 291)	115 (107 to 123)	232 (142 to 302)	120 (32 to 148)	123 (112 to 133)

RT – radiotherapy; EVRT – external beam radiotherapy; LDR – low dose rate; HDR – high dose rate; C-ions – carbon ions; BED – biologically equivalent dose, alpha / beta = 10.

Demographics and clinical characteristics of the lung cancer cohorts.

Characteristic		REQUITE N = 483	RADIOGEN N= 155
Age treatment	*Mean (range)*	70 (39 - 91)	64.2
Gender	*Male*	336	134
	Female	147	21
COPD	*Yes*	202	30
	No	281	125
Chemotherapy	*Yes*	248	144
	No	235	11
Radiotherapy technique	*3D-CRT*	157	155
	ARC	62	0
	IMRT	120	0
	Tomotherapy	11	0
	SABR	133	0
Prescription dose (BED)	*Mean (range)*	87.6 (11 – 190)	-
Smoker	*Never*	21	15
	Ex-smoker	267	91
	Current	108	47
FEV1 (litres)	*Mean (range)*	2 (0.5 – 4.2)	2.2
V20 lung (%)	*Mean (range)*	16.6 (0.1 - 99)	20.9
V35 Oesophagus (%)	*Mean (range)*	33.5 (0 - 100)	33.0
Diabetes	*Yes*	84	-
	No	399	
Diagnosis of RA	*Yes*	16	-
	No	467	

COPD – chronic obstructive pulmonary disorder; CRT – conformal radiotherapy; IMRT – intensity modulated radiotherapy; SABR – stereotactic ablative body radiotherapy; BED – biologically equivalent dose, alpha / beta = 10; FEV – forced expiratory volume; RA – Rheumatoid arthritis.

Demographics and clinical characteristics of the breast cancer cohorts.

**Characteristic**		**REQUITE**<br><br>**N = 1820**	**Cambridge IMRT trial**<br>**N = 910**
Age treatment	*Mean (range)*	58 (23 – 90)	58.7
Smoker	*Never*	1011	126
	Ex-smoker / current	788	776
Chemotherapy	*Yes*	559	151
	No	1252	749
Cardiovasculature disease	*Yes*	130	86
	No	1689	798
BMI	*Mean (range)*	26.3 (13.1 – 61.8)	27.4
Breast treatment volume (cm^3)	*Mean (range)*	786 (38 – 3839)	1145.9
Prescription dose (BED)	*Mean (range)*	56 (44.7 – 67.2)	-
Diabetes	*Yes*	100	-
	No	1720	
Post op infection	*Yes*	77	175
	No	1704	716
Breast boost	*Yes*	539	573
	No	1281	337
Diagnosis of RA	*Yes*	55	-
	No	1765	

BMI – body mass index; BED – biologically equivalent dose, alpha / beta = 10; RA – Rheumatoid arthritis.

Supplementary tables 3

Toxicity incidence from the REQUITE cohort used for the primary analysis in this study.

Acute Toxicity Incidence of the Prostate Cancer Cohort

Variable, frequency (%)	N	Overall, N = 1,205[1]	No RA Diagnosis, N = 1,180[1]	RA Diagnosis, N = 25[1]	p-value[2]
**Urinary Frequency**	1,161				0.2
0		409 (35%)	400 (35%)	9 (36%)	
1		419 (36%)	411 (36%)	8 (32%)	
2		229 (20%)	226 (20%)	3 (12%)	
3		104 (9.0%)	99 (8.7%)	5 (20%)	
**Nocturia**	1,172				0.2
0		405 (35%)	395 (34%)	10 (42%)	
1		434 (37%)	422 (37%)	12 (50%)	
2		248 (21%)	247 (22%)	1 (4.2%)	
3		78 (6.7%)	77 (6.7%)	1 (4.2%)	
4		7 (0.6%)	7 (0.6%)	0 (0%)	
**Urinary Urgency**	1,154				>0.9
0		463 (40%)	451 (40%)	12 (50%)	
1		244 (21%)	239 (21%)	5 (21%)	
2		163 (14%)	161 (14%)	2 (8.3%)	
3		174 (15%)	171 (15%)	3 (13%)	
4		110 (9.5%)	108 (9.6%)	2 (8.3%)	
**Hematuria**	1,143				0.3
		1,123 (98%)	1,100 (98%)	23 (96%)	
		20 (1.7%)	19 (1.7%)	1 (4.2%)	
**Urinary Incontinence**	1,166				0.8
0		836 (72%)	818 (72%)	18 (75%)	
1		141 (12%)	137 (12%)	4 (17%)	
2		94 (8.1%)	92 (8.1%)	2 (8.3%)	
3		63 (5.4%)	63 (5.5%)	0 (0%)	
4		32 (2.7%)	32 (2.8%)	0 (0%)	
**Decreased Urinary Stream**	1,153				0.7
0		778 (67%)	759 (67%)	19 (76%)	
1		326 (28%)	320 (28%)	6 (24%)	
2		45 (3.9%)	45 (4.0%)	0 (0%)	
3		4 (0.3%)	4 (0.4%)	0 (0%)	
**Dysuria**	1,152				0.2
0		604 (52%)	594 (53%)	10 (43%)	

1		383 (33%)	372 (33%)	11 (48%)	
2		103 (8.9%)	102 (9.0%)	1 (4.3%)	
3		49 (4.3%)	49 (4.3%)	0 (0%)	
4		13 (1.1%)	12 (1.1%)	1 (4.3%)	
**Gastrointenstinal Incontinence**	1,154				0.13
0		930 (81%)	911 (81%)	19 (79%)	
1		130 (11%)	129 (11%)	1 (4.2%)	
2		55 (4.8%)	51 (4.5%)	4 (17%)	
3		28 (2.4%)	28 (2.5%)	0 (0%)	
4		11 (1.0%)	11 (1.0%)	0 (0%)	
**Diarrhoea**	1,160				0.15
0		829 (71%)	811 (71%)	18 (75%)	
1		268 (23%)	265 (23%)	3 (13%)	
2		49 (4.2%)	46 (4.0%)	3 (13%)	
3		14 (1.2%)	14 (1.2%)	0 (0%)	
**Gastrointenstinal Urgency**	1,154				0.6
0		524 (45%)	513 (45%)	11 (48%)	
1		252 (22%)	248 (22%)	4 (17%)	
2		178 (15%)	174 (15%)	4 (17%)	
3		158 (14%)	156 (14%)	2 (8.7%)	
4		42 (3.6%)	40 (3.5%)	2 (8.7%)	
**Tenesmus**	1,154				0.5
0		856 (74%)	838 (74%)	18 (75%)	
1		131 (11%)	128 (11%)	3 (13%)	
2		74 (6.4%)	72 (6.4%)	2 (8.3%)	
3		69 (6.0%)	69 (6.1%)	0 (0%)	
4		24 (2.1%)	23 (2.0%)	1 (4.2%)	
**Gastrointestinal Pain**	1,156				0.08
0		748 (65%)	734 (65%)	14 (61%)	
1		305 (26%)	298 (26%)	7 (30%)	
2		72 (6.2%)	72 (6.4%)	0 (0%)	
3		27 (2.3%)	26 (2.3%)	1 (4.3%)	
4		4 (0.3%)	3 (0.3%)	1 (4.3%)	
**Rectal Mucus**	1,154				0.2
0		733 (64%)	717 (64%)	16 (64%)	
1		175 (15%)	173 (15%)	2 (8.0%)	
2		172 (15%)	169 (15%)	3 (12%)	
3		56 (4.9%)	53 (4.7%)	3 (12%)	
4		18 (1.6%)	17 (1.5%)	1 (4.0%)	

**Constipation**	1,156				0.8
0		1,064 (92%)	1,042 (92%)	22 (92%)	
1		76 (6.6%)	74 (6.5%)	2 (8.3%)	
2		9 (0.8%)	9 (0.8%)	0 (0%)	
3		3 (0.3%)	3 (0.3%)	0 (0%)	
4		4 (0.3%)	4 (0.4%)	0 (0%)	
**STAT Acute**	1,180				>0.9
N		1,180	1,155	25	
Mean (SD)		0.00 (0.47)	0.00 (0.47)	-0.02 (0.40)	
Median (IQR)		-0.09 (-0.35, 0.24)	-0.09 (-0.35, 0.24)	-0.12 (-0.24, 0.10)	
Range		-0.74, 2.35	-0.74, 2.35	-0.58, 1.12	

[1] n (%)

[2] Wilcoxon rank sum test; Fisher's exact test

Late Toxicity Incidence of the Prostate Cancer Cohort

**Variable, frequency (%)**	**N**	**Overall, N = 1,494**[1]	**No RA Diagnosis, N = 1,465**[1]	**RA Diagnosis, N = 29**[1]	**p-value**[2]
**Urinary Frequency**	1,346				0.5
0		775 (58%)	761 (58%)	14 (52%)	
1		372 (28%)	365 (28%)	7 (26%)	
2		139 (10%)	134 (10%)	5 (19%)	
3		60 (4.5%)	59 (4.5%)	1 (3.7%)	
**Hematuria**	1,258				>0.9
0		1,179 (94%)	1,156 (94%)	23 (96%)	
1		79 (6.3%)	78 (6.3%)	1 (4.2%)	
**Decreased Urinary Stream**	1,338				0.9
0		987 (74%)	965 (74%)	22 (81%)	
1		310 (23%)	305 (23%)	5 (19%)	
2		33 (2.5%)	33 (2.5%)	0 (0%)	
3		8 (0.6%)	8 (0.6%)	0 (0%)	
**Rectal Bleeding**	1,255				>0.9
0		1,031 (82%)	1,009 (82%)	22 (81%)	
1		224 (18%)	219 (18%)	5 (19%)	
**Rectal Mucus**	1,344				0.12
0		884 (66%)	870 (66%)	14 (50%)	
1		226 (17%)	217 (16%)	9 (32%)	
2		179 (13%)	176 (13%)	3 (11%)	
3		42 (3.1%)	40 (3.0%)	2 (7.1%)	
4		13 (1.0%)	13 (1.0%)	0 (0%)	
**STAT Late**	1,407				>0.9

N	1,407	1,379	28	
Mean (SD)	0.02 (0.59)	0.02 (0.58)	0.07 (0.68)	
Median (IQR)	-0.08 (-0.52, 0.31)	-0.08 (-0.52, 0.31)	-0.22 (-0.30, 0.33)	
Range	-0.73, 3.87	-0.73, 3.87	-0.52, 2.04	

[1] n (%)

[2] Wilcoxon rank sum test; Fisher's exact test

Acute Toxicity Incidence of the Lung Cancer Cohort

Variable, frequency (%)	N	Overall, N = 412[1]	No RA Diagnosis, N = 396[1]	RA Diagnosis, N = 16[1]	p-value[2]
**Cough**	407				0.1
0		188 (46%)	179 (46%)	9 (56%)	
1		186 (46%)	182 (47%)	4 (25%)	
2		33 (8.1%)	30 (7.7%)	3 (19%)	
**Dyspnoea**	406				0.4
0		154 (38%)	145 (37%)	9 (56%)	
1		166 (41%)	162 (42%)	4 (25%)	
2		70 (17%)	68 (17%)	2 (13%)	
3		15 (3.7%)	14 (3.6%)	1 (6.3%)	
4		1 (0.2%)	1 (0.3%)	0 (0%)	
**Pneumonitis**	407				0.6
0		350 (86%)	337 (86%)	13 (81%)	
1		35 (8.6%)	33 (8.4%)	2 (13%)	
2		18 (4.4%)	17 (4.3%)	1 (6.3%)	
3		4 (1.0%)	4 (1.0%)	0 (0%)	
**Dysphagia**	411				0.7
0		354 (86%)	340 (86%)	14 (88%)	
1		37 (9.0%)	36 (9.1%)	1 (6.3%)	
2		16 (3.9%)	15 (3.8%)	1 (6.3%)	
3		4 (1.0%)	4 (1.0%)	0 (0%)	
**Oesophagitis**	411				0.4
0		364 (89%)	350 (89%)	14 (88%)	
1		22 (5.4%)	22 (5.6%)	0 (0%)	
2		20 (4.9%)	18 (4.6%)	2 (13%)	
3		5 (1.2%)	5 (1.3%)	0 (0%)	
**STAT Acute**	412				0.6
N		412	396	16	
Mean (SD)		0.00 (0.59)	0.00 (0.58)	-0.01 (0.81)	
Median (IQR)		-0.06 (-0.38, 0.24)	-0.06 (-0.38, 0.24)	-0.16 (-0.62, 0.20)	

Range		-0.62, 2.68	-0.62, 2.68	-0.62, 2.68	

[1] n (%)

[2] Fisher's exact test; Wilcoxon rank sum test

Late Toxicity Incidence of the Lung Cancer Cohort

**Variable, frequency (%)**	**N**	**Overall, N = 367[1]**	**No RA Diagnosis, N = 352[1]**	**RA Diagnosis, N = 15[1]**	**p-value[2]**
**Cough**	361				**0.047**
0		115 (32%)	108 (31%)	7 (50%)	
1		188 (52%)	185 (53%)	3 (21%)	
2		58 (16%)	54 (16%)	4 (29%)	
**Dyspnoea**	362				0.09
0		88 (24%)	82 (24%)	6 (40%)	
1		134 (37%)	132 (38%)	2 (13%)	
2		119 (33%)	114 (33%)	5 (33%)	
3		21 (5.8%)	19 (5.5%)	2 (13%)	
**Pneumonitis**	360				0.3
0		270 (75%)	257 (74%)	13 (93%)	
1		53 (15%)	53 (15%)	0 (0%)	
2		36 (10%)	35 (10%)	1 (7.1%)	
3		1 (0.3%)	1 (0.3%)	0 (0%)	
**Dysphagia**	364				0.4
0		316 (87%)	303 (87%)	13 (93%)	
1		32 (8.8%)	32 (9.1%)	0 (0%)	
2		15 (4.1%)	14 (4.0%)	1 (7.1%)	
3		1 (0.3%)	1 (0.3%)	0 (0%)	
**Oesophagitis**	364				0.7
0		334 (92%)	321 (92%)	13 (93%)	
1		19 (5.2%)	18 (5.1%)	1 (7.1%)	
2		11 (3.0%)	11 (3.1%)	0 (0%)	
**STAT Late**	367				0.4
N		367	352	15	
Mean (SD)		0.02 (0.60)	0.02 (0.60)	-0.06 (0.67)	
Median (IQR)		-0.06 (-0.37, 0.30)	-0.01 (-0.31, 0.30)	-0.30 (-0.61, 0.41)	
Range		-0.76, 2.67	-0.76, 2.67	-0.76, 1.18	

[1] n (%)

[2] Wilcoxon rank sum test; Fisher's exact test

Acute Toxicity Incidence of the Breast Cancer Cohort

Variable, frequency (%)	N	Overall, N = 1,067[1]	No RA Diagnosis, N = 1,038[1]	RA Diagnosis, N = 29[1]	p-value[2]
**Erythema**	1,058				0.4
0		155 (15%)	153 (15%)	2 (6.9%)	
1		615 (58%)	598 (58%)	17 (59%)	
2		269 (25%)	260 (25%)	9 (31%)	
3		19 (1.8%)	18 (1.7%)	1 (3.4%)	
**Ulceration**	1,053				>0.9
0		927 (88%)	901 (88%)	26 (90%)	
1		82 (7.8%)	80 (7.8%)	2 (6.9%)	
2		36 (3.4%)	35 (3.4%)	1 (3.4%)	
3		8 (0.8%)	8 (0.8%)	0 (0%)	
**STAT Acute**	1,062				0.5
N		1,062	1,033	29	
Mean (SD)		-0.01 (0.78)	-0.02 (0.78)	0.08 (0.75)	
Median (IQR)		-0.28 (-0.28, 0.45)	-0.28 (-0.28, 0.45)	-0.28 (-0.28, 0.45)	
Range		-1.72, 4.13	-1.72, 4.13	-1.02, 2.41	

[1] n (%)
[2] Fisher's exact test;
Wilcoxon rank sum test

Late Toxicity Incidence of the Breast Cancer Cohort

Variable, frequency (%)	N	Overall, N = 1,820[1]	No RA Diagnosis, N = 1,765[1]	RA Diagnosis, N = 55[1]	p-value[2]
**Telangiectasia**	1,740				0.4
0		1,625 (93%)	1,578 (93%)	47 (90%)	
1		106 (6.1%)	101 (6.0%)	5 (9.6%)	
2		9 (0.5%)	9 (0.5%)	0 (0%)	
**Induration**	1,739				0.9
0		1,337 (77%)	1,298 (77%)	39 (75%)	
1		354 (20%)	342 (20%)	12 (23%)	
2		44 (2.5%)	43 (2.5%)	1 (1.9%)	

3		4 (0.2%)	4 (0.2%)	0 (0%)	
**Pigmentation**	1,740				0.14
0		1,099 (63%)	1,062 (63%)	37 (71%)	
1		629 (36%)	615 (36%)	14 (27%)	
2		12 (0.7%)	11 (0.7%)	1 (1.9%)	
**Atrophy**	1,734				0.3
0		984 (57%)	958 (57%)	26 (50%)	
1		587 (34%)	569 (34%)	18 (35%)	
2		142 (8.2%)	134 (8.0%)	8 (15%)	
3		21 (1.2%)	21 (1.2%)	0 (0%)	
**Oedema**	1,750				0.02
0		1,356 (77%)	1,316 (78%)	40 (77%)	
1		331 (19%)	325 (19%)	6 (12%)	
2		62 (3.5%)	56 (3.3%)	6 (12%)	
3		1 (<0.1%)	1 (<0.1%)	0 (0%)	
**STAT Late**	1,753				>0.9
N		1,753	1,701	52	
Mean (SD)		0.01 (0.54)	0.01 (0.54)	0.02 (0.54)	
Median (IQR)		-0.09 (-0.32, 0.25)	-0.09 (-0.32, 0.25)	-0.14 (-0.32, 0.42)	
Range		-0.56, 3.57	-0.56, 3.57	-0.53, 1.83	

[1] n (%)

[2] Wilcoxon rank sum test; Fisher's exact test

Supplementary figures 1 – STAT distributions

The distributions of STAT are included here for the REQUITE cohort as the primary discovery cohort. As is expected, the distributions show a right-sided skew as a small number of individuals will experience more severe toxicity.

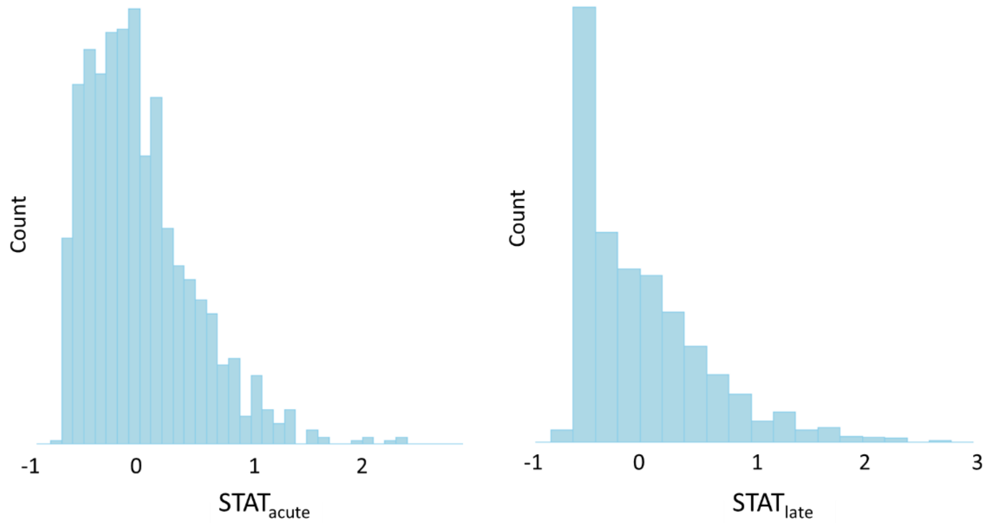

Prostate $STAT_{acute}$ – toxicities included: Urinary frequency, Nocturia, Urinary urgency, Haematuria, Urinary incontinence, Decreased stream, Dysuria,

Gastrointestinal incontinence, Diarrhoea, Gastrointestinal urgency, Tenesmus, Gastrointestinal pain, Rectal mucus, Constipation.

Prostate $STAT_{late}$ – toxicities included: Proctitis, Rectal bleeding, Haematuria, Urinary frequency, Urinary retention.

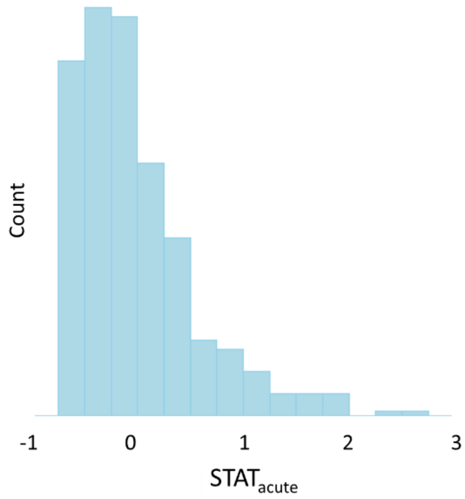

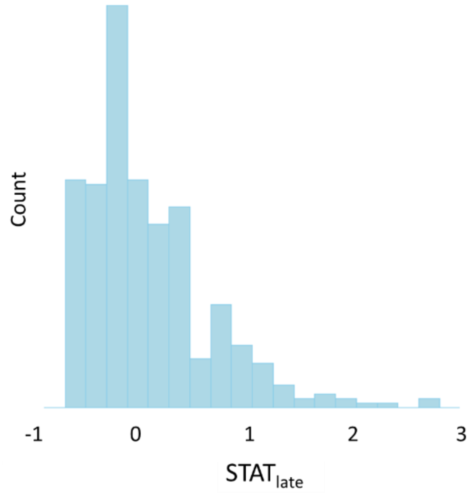

Lung $STAT_{acute}$ – toxicities included: Cough, Dyspnoea, Pneumonitis, Dysphagia, Oesophagitis.

Lung $STAT_{late}$ – toxicities included: Cough, Dyspnoea, Pneumonitis, Dysphagia, Oesophagitis.

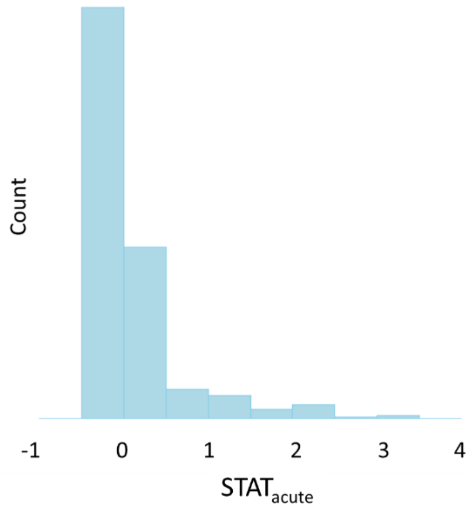

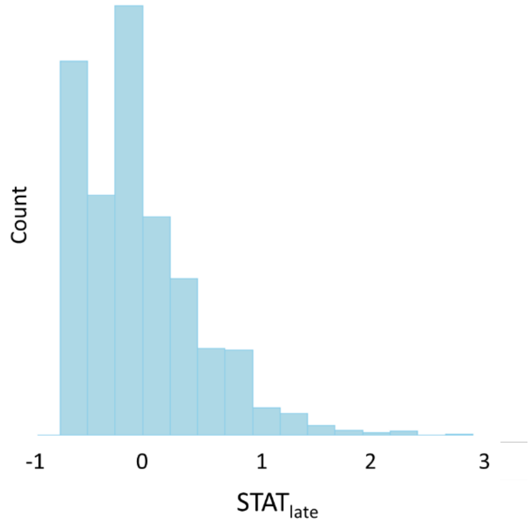

Breast $STAT_{acute}$ – toxicities included: Erythema, Ulceration

Breast $STAT_{late}$ – toxicities included: Telangiectasia, Oedema, Induration, Pigmentation, Atrophy

Supplementary tables 4 – weighted-PRS STAT acute and STAT late results

	$STAT_{acute}$ (wPRS continuous)				$STAT_{acute}$ (wPRS 90th percentile)			
	Univariable		Multivariable		Univariable		Multivariable	
	Beta (95% CI)	p	Beta (95% CI)	p	Beta (95% CI)	p	Beta (95% CI)	p
Prostate	-0.015 (-0.044, 0.014)	0.31	-0.017 (-0.045, 0.012)	0.25	-0.091 (-0.180, -0.002)	0.05	-0.086 (-0.173, 0.001)	0.05
Lung	-0.009 (-0.073, 0.058)	0.82	-0.011 (-0.074, 0.053)	0.74	-0.027 (-0.223, 0.170)	0.79	-0.012 (-0.021, 0.172)	0.85
Breast	0.001 (-0.049, 0.050)	0.98	0.011 (-0.060, 0.038)	0.66	-0.095 (-0.248, 0.249)	0.23	-0.100 (-0.250, 0.052)	0.20

	$STAT_{late}$ (wPRS continuous)				$STAT_{late}$ (wPRS 90th percentile)			
	Univariable		Multivariable		Univariable		Multivariable	
	Beta (95% CI)	p	Beta (95% CI)	p	Beta (95% CI)	p	Beta (95% CI)	p
Prostate	0.015 (0.018, 0.049)	0.37	0.015 (-0.019, 0.049)	0.39	-0.065 (-0.166, 0.037)	0.21	-0.065 (-0.166, 0.037)	0.21
Lung	0.023 (-0.044, 0.090)	0.51	0.028 (-0.039, 0.095)	0.42	0.170 (-0.032, 0.370)	0.10	0.120 (-0.004, 0.396)	0.06
Breast	-0.014 (-0.041, 0.012)	0.31	-0.008 (-0.034, 0.018)	0.53	-0.057 (-0.141, 0.027)	0.19	-0.028 (-0.109, 0.054)	0.51

Univariable and multivariable analysis for acute STAT and late STAT with wprs as a continuous variable and dichotomised at the 90th percentile. For the multivariable analysis the following adjustment variables were included:

- Prostate: age at radiotherapy, diabetes, prior surgery, hormone therapy, prescription dose (converted to BED);
- Lung: gender, age at radiotherapy, prior/current smoker, radiotherapy technique (3D-conformal, arc, IMRT, tomotherapy, Strerotactic radiotherapy), fev1, v20 lungs, v35 oesophagus, prescription dose (converted to BED), diagnosis of COPD;
- Breast: age at radiotherapy, prior/current smoker, history of cardio-vasculature disease, BMI, breast volume, diagnosis of diabetes, post-operative breast infection, received breast boost.

Supplementary tables 5 – Verification results
Prostate

Distribution of the PRS and wPRS in the full Radiogenomics Consortium (RGC) prostate datasets available for validation, patient characteristics described in S3 and toxicity definitions in S1. The distributions are closely match those calculated for the REQUITE cohort and included in figure 1.

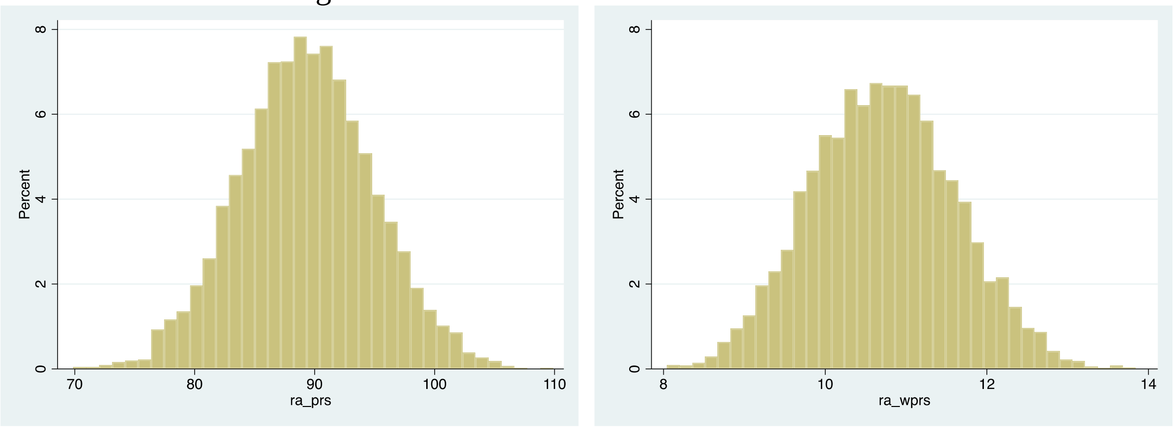

Association of $STAT_{acute}$ and $STAT_{late}$ with the PRS and wPRS as continuous and for the top 10th percentile. No significant associations were found on univariable or multivariable analysis. Here, participants – univariable analyses are adjusted for cohort (to adjust for demographics across cohorts); multivariable analyses are adjusted for age, totalBED, ADT, surgery, diabetes, and cohort. Two analysis were performed; (1) for European only ancestry and (2) for the full RGC cohort.

(1) European ancestry

	Univariable[a]		Multivariable[b]	
	Beta (95% CI)	p	Beta (95% CI)	p
$STAT_{acute}$ (PRS continuous)	0.001646	0.47	0.000931	0.68
$STAT_{acute}$ (PRS percentile)	0.062166	0.16	0.044935	0.32
$STAT_{acute}$ (wPRS continuous)	-0.003442	0.81	-0.008569	0.55
$STAT_{acute}$ (wPRS percentile)	0.017629	0.67	-0.017720	0.68
$STAT_{late}$ (PRS continuous)	0.000557	0.73	0.000811	0.63
$STAT_{late}$ (PRS percentile)	-0.005158	0.87	-0.000847	0.98
$STAT_{late}$ (wPRS continuous)	0.001564	0.88	0.002640	0.80
$STAT_{late}$ (wPRS percentile)	-0.024403	0.42	-0.029699	0.34

[a] $STAT_{acute}$ univariable analysis included 2,816 participants and $STAT_{late}$ univariable analysis included 4,264 participants
[b] $STAT_{acute}$ multivariable analysis included 2,683 participants and $STAT_{late}$ multivariable analysis included 4,006 participants

(2) Full RGC cohorts

	Univariable[a]		Multivariable[b]	
	Beta (95% CI)	p	Beta (95% CI)	p
$STAT_{acute}$ (PRS continuous)	0.000688	0.71	0.000829	0.71
$STAT_{acute}$ (PRS percentile)	0.038826	0.27	0.036802	0.40
$STAT_{acute}$ (wPRS continuous)	-0.007341	0.53	-0.011062	0.43
$STAT_{acute}$ (wPRS percentile)	0.005377	0.87	-0.026592	0.53
$STAT_{late}$ (PRS continuous)	0.001612	0.26	0.000682	0.67
$STAT_{late}$ (PRS percentile)	0.021822	0.41	0.010324	0.73
$STAT_{late}$ (wPRS continuous)	0.006118	0.50	0.001442	0.89
$STAT_{late}$ (wPRS percentile)	-0.012931	0.63	-0.031011	0.31

[a] $STAT_{acute}$ univariable analysis included 3,831 participants and $STAT_{late}$ univariable analysis included 5,441 participants
[b] $STAT_{acute}$ multivariable analysis included 2,749 participants and $STAT_{late}$ multivariable analysis included 4,208 participants

Finally, association between RA PRS and wPRS as continuous and for the top 10th percentile for individual toxicity endpoints. Multivariable analysis with adjustment variables as defined in the STAT analysis above. No association was found between a high genetic risk and any individual end-points.

	**Urinary frequency-PRO N = 2,868**	**Urinary frequency-Obs N = 3,194**	**Hematuria-PRO N = 2,287**	**Hematuria-Obs N = 3,946**
**RA-PRS**	0.0021 (-0.0043 to 0.0086) p = 0.52	0.0049 (-0.0013 to 0.0111) p = 0.12	0.0008 (-0.0061 to 0.0078) p = 0.81	0.0008 (-0.0046 to 0.0063) p = 0.76
**RA-wPRS**	-0.0014 (-.0042 to 0.0395) p = 0.95	0.0176 (-0.0211 to 0.0563) p = 0.37	0.0022 (-0.0420 to 0.0464) p = 0.92	0.0019 (-0.0324 to 0.0362) p = 0.91
**RA-PRS 90th percentile**	-0.0232 (-0.1947 to 0.1484) p = 0.79	0.0051 (-0.1636 to 0.1737) p = 0.95	-0.0205 (-0.2063 to 0.1652) p = 0.83	0.0273 (-0.1184 to 0.1731) p = 0.71
**RA-wPRS 90th percentile**	0.0043 (-0.1616 to 0.1703) p = 0.96	-0.0321 (-0.1803 to 0.1162) p = 0.67	0.0113 (-0.1585 to 0.1811) p = 0.90	-0.0576 (-0.1957 to 0.0806) p = 0.41

Lung

Validation analysis for the RADIOGEN_Lung cohort, patient characteristics in S3 and toxicity definitions in S1, 155 patient were available in the cohort.

Multivariable analysis of acute toxicity endpoints and $STAT_{acute}$. No results were statistically significant after Bonferroni correction for multiple comparisons. Adjustment variables were match to the primary analysis: sex, age, smoking status, radiotherapy technique, fev1, v20 lungs, v35 oesophagus, prescription dose (BED, alpha / beta = 10), COPD.

	Continuous		90th Percentile	
	Beta	p	Beta	p
STAT acute	PRS: 0.006<br>wPRS: 0.037	0.43<br>0.49	PRS: -0.118<br>wPRS: 0.140	0.35<br>0.33
Cough	PRS: -0.022<br>wPRS: -0.098	0.01<br>0.12	PRS: -0.301<br>wPRS: -0.177	0.03<br>0.29
Dyspnoea	PRS: 0.026<br>wPRS: 0.117	0.08<br>0.24	PRS: 0.007<br>wPRS: 0.522	0.98<br>0.06
Pneumonitis	PRS: 0.003<br>wPRS: 0.043	0.72<br>0.49	PRS: 0.075<br>wPRS: 0.025	0.62<br>0.89
Dysphagia	PRS: 0.018<br>wPRS: 0.076	0.09<br>0.29	PRS: 0.010<br>wPRS: 0.161	0.95<br>0.42
Oesophagitis	PRS: 0.004<br>wPRS: 0.027	0.70<br>0.72	PRS: -0.182<br>wPRS: 0.051	0.30<br>0.80

Multivariable analysis of late toxicity endpoints and $STAT_{late}$. No results were statistically significant after Bonferroni correction for multiple comparisons. Adjustment variables were match to the primary analysis: sex, age, smoking status, radiotherapy technique, fev1, v20 lungs, v35 oesophagus, prescription dose (BED, alpha / beta = 10), COPD.

	Continuous		90th Percentile	
	Beta	p	Beta	p
STAT late	PRS: -0.007	0.49	PRS: -0.086	0.62
	wPRS: -0.013	0.86	wPRS: 0.005	0.98
Cough	PRS: -0.011	0.27	PRS: 0.043	0.79
	wPRS: -0.053	0.44	wPRS: -0.087	0.64
Dyspnoea	PRS: 0.015	0.45	PRS: 0.106	0.74
	wPRS: 0.048	0.72	wPRS: 0.272	0.46
Pneumonitis	PRS: 0.001	0.97	PRS: 0.044	0.82
	wPRS: 0.027	0.74	wPRS: -0.177	0.44
Dysphagia	PRS: -0.013	0.24	PRS: -0.292	0.10
	wPRS: -0.034	0.65	wPRS: 0.002	0.99
Oesophagitis	PRS: -0.004	0.68	PRS: -0.041	0.79
	wPRS: 0.009	0.89	wPRS: 0.047	0.79

Breast

Validation analysis of the Cambridge IMRT cohort, patient characteristics in S3 and toxicity definitions in S1, 910 patient were available in the cohort.

The calculated distribution of the PRS shows close agreement with the REQUITE dataset calculation included in figure 1.

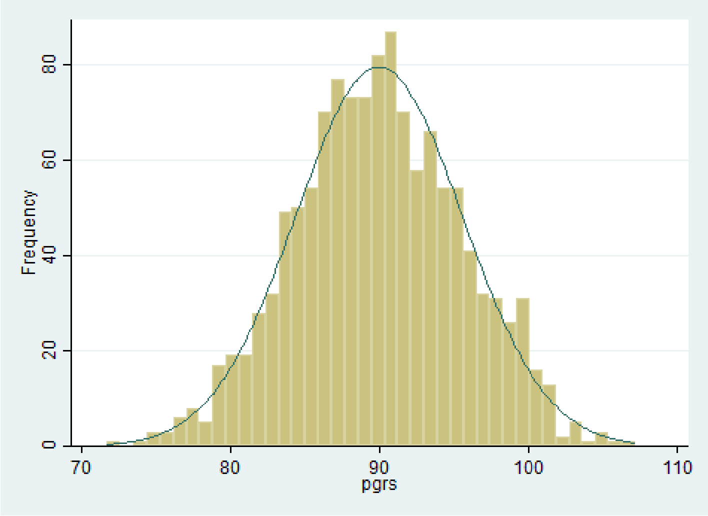

$STAT_{acute}$ showed no association with the PRS, coefficient 0.007 (-0.016,0.030), p = 0.56.

Results from the multivariable analysis is included below for $STAT_{late}$ and individual late toxicity endpoints. No significant results were found. The multivariable analysis was matched to the primary analysis with the adjustment variables: age at radiotherapy, prior/current smoker, history of cardio-vasculature disease, BMI, breast volume, diagnosis of diabetes, post-operative breast infection, received breast boost.

	Univariable		Multivariable	
	beta (95% CI)	p	beta (95% CI)	p
STAT-late	-0.003 (-0.009, 0.003)	0.35	-0.001 (-0.012, 0.010)	0.85
Telangiectasia	-0.005 (-0.34, 0.023)	0.71	0.002 (-0.010, 0.014)	0.77
Oedema	0.003 (-0.020, 0.025)	0.82	0.004 (-0.008,0.015)	0.54
Induration	-0.016 (-0.036, 0.004)	0.12	-0.002 (-0.013, 0.009)	0.74
Pigmentation	-0.018 (-0.052, 0.14)	0.28	-0.003 (-0.016, 0.010)	0.66
Pain	-0.015 (-0.039, 0.009)	0.21	-0.006 (-0.018, 0.006)	0.31
Oversensitivity	-0.008 (-0.032, 0.017)	0.54	-0.004 (-0.017, 0.009)	0.53

Supplementary tables 5 – Diagnosis-based analysis results

	$STAT_{acute}$ (RA diagnosis)						$STAT_{late}$ (RA diagnosis)					
	Univariable			*Multivariable*			*Univariable*			*Multivaraible*		
	Beta	(95% CI)	p	Beta	(95% CI)	p	Beta	(95% CI)	p	Beta	(95% CI)	p
Prostate	-0.02	(-0.21, 0.16)	0.81	-0.02	(-0.20, 0.17)	0.86	0.05	(-0.17, 0.27)	0.66	0.05	(-0.17, 0.27)	0.66
Lung	-0.01	(-0.30, 0.29)	0.96	0.08	(-0.24, 0.40)	0.63	-0.08	(-0.39, 0.23)	0.6	-0.09	(-0.42, 0.24)	0.60
Breast	0.1	(-0.19, 0.38)	0.51	-0.02	(-0.30, 0.26)	0.91	0.01	(-0.13, 0.16)	0.86	-0.08	(-0.22, 0.07)	0.28

Univariable and multivariable analysis for $STAT_{acute}$ and $STAT_{late}$ by dichotomized RA diagnosis. For the multivariable analysis the following adjustment variables were included, prostate: age at radiotherapy, diabetes, prior surgery, hormone therapy, prescription dose (converted to BED); lung: sex, age at radiotherapy, prior/current smoker, radiotherapy technique (3D-conformal, arc, IMRT, tomotherapy, stereotactic radiotherapy), fev1, v20 lungs, v35 oesophagus, prescription dose (converted to BED), diagnosis of COPD; breast: age at radiotherapy, prior/current smoker, history of cardio-vasculature disease, BMI, breast volume, diagnosis of diabetes, post-operative breast infection, received breast boost.

References

1. Seibold P, Webb A, Aguado-Barrera ME, Azria D, Bourgier C, Brengues M, et al. REQUITE: A prospective multicentre cohort study of patients undergoing radiotherapy for breast, lung or prostate cancer. Radiother Oncol 2019;138:59–67.
2. West C, Azria D, Chang-Claude J, Davidson S, Lambin P, Rosenstein B, et al. The REQUITE Project: Validating Predictive Models and Biomarkers of Radiotherapy Toxicity to Reduce Side-effects and Improve Quality of Life in Cancer Survivors. Clin Oncol 2014;26:739–42.
3. Dearnaley DP, Sydes MR, Graham JD, Aird EG, Bottomley D, Cowan RA, et al. Escalated-dose versus standard-dose conformal radiotherapy in prostate cancer: first results from the MRC RT01 randomised controlled trial. Lancet Oncol 2007;8:475–87.
4. Dearnaley D, Syndikus I, Mossop H, Khoo V, Birtle A, Bloomfield D, et al. Conventional versus hypofractionated high-dose intensity-modulated radiotherapy for prostate cancer: 5-year outcomes of the randomised, non-inferiority, phase 3 CHHiP trial. Lancet Oncol 2016;17:1047–60.
5. West C, Rosenstein BS, Alsner J, Azria D, Barnett G, Begg A, et al. Establishment of a Radiogenomics Consortium. Int J Radiat Oncol Biol Phys. 2010 Apr;76(5):1295-6.
6. Mukesh MB, Barnett GC, Wilkinson JS, Moody AM, Wilson C, Dorling L, et al. Randomized controlled trial of intensity-modulated radiotherapy for early breast cancer: 5-year results confirm superior overall cosmesis. J Clin Oncol Off J Am Soc Clin Oncol 2013;31:4488–95.
7. Fachal L, Gómez-Caamaño A, Barnett GC, Peleteiro P, Carballo AM, Calvo-Crespo P, et al. A three-stage genome-wide association study identifies a susceptibility locus for late radiotherapy toxicity at 2q24.1. Nat Genet 2014;46:891–4.

8. Aguado-Barrera ME, Martínez-Calvo L, Fernández-Tajes J, Calvo-Crespo P, Taboada-Valladares B, Lobato-Busto R, et al. Validation of Polymorphisms Associated with the Risk of Radiation-Induced Oesophagitis in an Independent Cohort of Non-Small-Cell Lung Cancer Patients. Cancers 2021, Vol 13, Page 1447 2021;13:1447.
